# Cancer-specific tissue-resident memory T-cells express ZNF683 in colorectal cancer

**DOI:** 10.1038/s41416-023-02202-4

**Published:** 2023-03-03

**Authors:** Masatoshi Kitakaze, Mamoru Uemura, Tomoaki Hara, Ryota Chijimatsu, Daisuke Motooka, Toshiro Hirai, Masamitsu Konno, Daisuke Okuzaki, Yuki Sekido, Tsuyoshi Hata, Takayuki Ogino, Hidekazu Takahashi, Norikatsu Miyoshi, Ken Ofusa, Tsunekazu Mizushima, Hidetoshi Eguchi, Yuichiro Doki, Hideshi Ishii

**Affiliations:** 1 Department of Gastroenterological Surgery, Osaka University Graduate School of Medicine, Suita, Osaka 565-0871 Japan; 2 Department of Medical Data Science, Center of Medical Innovation and Translational Research, Osaka University Graduate School of Medicine, Suita, Osaka 565-0871 Japan; 3 Genome Information Research Center, Research Institute for Microbial Diseases, Osaka University, Suita, Osaka 565-0871 Japan; 4 Prophoenix Division, Food and Life-Science Laboratory, Idea Consultants, Inc., Osaka-city, Osaka 559-8519 Japan; 5 Present Address: National Institute of Advanced Industrial Science and Technology, Koto-ku, Tokyo 135-0064 Japan

**Keywords:** Colon cancer, Rectal cancer, Cancer microenvironment

## Abstract

**Background:**

Tissue-resident memory T (Trm) cells are associated with cytotoxicity not only in viral infection and autoimmune disease pathologies but also in many cancers. Tumour-infiltrating CD103^+^ Trm cells predominantly comprise CD8 T cells that express cytotoxic activation and immune checkpoint molecules called exhausted markers. This study aimed to investigate the role of Trm in colorectal cancer (CRC) and characterise the cancer-specific Trm.

**Methods:**

Immunochemical staining with anti-CD8 and anti-CD103 antibodies for resected CRC tissues was used to identify the tumour-infiltrating Trm cells. The Kaplan–Meier estimator was used to evaluate the prognostic significance. Cells immune to CRC were targeted for single-cell RNA-seq analysis to characterise cancer-specific Trm cells in CRC.

**Results:**

The number of CD103^+^/CD8^+^ tumour-infiltrating lymphocytes (TILs) was a favourable prognostic and predictive factor of the overall survival and recurrence-free survival in patients with CRC. Single-cell RNA-seq analysis of 17,257 CRC-infiltrating immune cells revealed a more increased zinc finger protein 683 (ZNF683) expression in cancer Trm cells than in noncancer Trm cells and in high-infiltrating Trm cells than low-infiltrating Trm in cancer, with an upregulated T-cell receptor (TCR)- and interferon-γ (IFN-γ) signalling-related gene expression in ZNF683^+^ Trm cells.

**Conclusions:**

The number of CD103^+^/CD8^+^ TILs is a prognostic predictive factor in CRC. In addition, we identified the ZNF683 expression as one of the candidate markers of cancer-specific Trm cells. IFN-γ and TCR signalling and ZNF683 expression are involved in Trm cell activation in tumours and are promising targets for cancer immunity regulation.

## Background

The immune system primarily functions by discriminating between “self” and “non-self” [1] cells, such as infected or cancerous cells. Immunity has been well-known to suppress cancer growth since Ehrlich et al. first predicted in 1909 that the immune system represses cancer growth [2]. Among immune cells, CD8^+^ T cells play an essential role in cancer immunity. Infiltrating CD8^+^ T cells represent a favourable prognostic marker in many solid tumours [3].

Recently, CD103^+^ CD8^+^ T cells were found to play an important role in cancer immunity, particularly in cancers of epithelial origin [3]. Among tumours with similar T-cell infiltration degrees, those with the greatest CD103^+^ CD8^+^ T-cell proportion have the best prognosis [4]. CD103^+^ CD8^+^ T cells, known as tissue-resident memory T (Trm) cells, are a unique subset of memory T cells [5]. Traditionally, memory T cells are categorised as central memory T (Tcm) cells or effector memory T (Tem) cells, according to the homing receptor expression [6]. Trm cells remain in peripheral tissues for a long time [7]. Binding of CD103 to E-cadherin, which is expressed on epithelial cells, allows Trm cells to be maintained in the local tissue microenvironment and is believed to be strongly involved in local immunity [8]. Trm cells have further been associated with cytotoxicity in the pathologies of viral infections and autoimmune diseases. Since the earliest discovery of Trm cells in the peripheral tissues of mice infected with a virus or bacterium, such as vesicular stomatitis virus or *Listeria monocytogenes* [9], Trm cells have also been reported to contribute to the pathology of autoimmune and inflammatory diseases, such as multiple sclerosis [10], type 1 diabetes mellitus [11], and inflammatory bowel disease [12]. Trm cells were also localised in solid tumours and played a role in inhibiting cancer progression and metastasis.

Trm cells may be involved in cancer immunity from the time of cancer cell development by residing in epithelial parts of peripheral tissues [13]. They are associated with favourable outcomes in patients with oropharyngeal squamous cell carcinoma, head and neck squamous cell carcinoma, breast cancer, non-small-cell lung cancer, bladder cancer, and melanoma [12, 14–26]. Trm cells can release effector molecules and cytokines, such as interleukin-2, interferon-gamma (IFN-γ), tumour necrosis factor-α, and granzyme B. Sustained high expression of these factors may enable Trm cells to rapidly and strongly respond against cancer cells [24–31].

Trm cell infiltration is a prognostic factor in many carcinomas, possibly because they play an important role in tumour immunity. However, not all Trm cells respond to cancer cells; therefore, distinguishing between bystander Trm cells, which recognise a wide range of epitopes unrelated to cancer, and cancer-specific cells is important [32]. Thus identifying and characterising cancer-specific Trm cells is vital to understand their role in cancer immunity.

This study investigated the association between Trm cell infiltration and good prognosis in patients with CRC and further characterised cancer-specific Trm cells in CRC at the single-cell level. We focused on zinc finger protein 683 (ZNF683) as a cancer-specific Trm cell marker and analysed its expression from single-cell RNA-seq analysis of 17,257 colorectal cancer (CRC)-infiltrating immune cells. Furthermore, we validated the CRC-infiltrating immune cells of 59,364 cells in 39 cases from public data and confirmed the same results as our data. Our findings provide new insights into the use of Trm cells as a predictive factor of CRC prognosis, ZNF683 as a candidate marker for cancer-specific Trm, and the characteristics of ZNF683^+^ Trm.

## Methods

### Study design and patients

This study evaluated 126 patients with CRC who underwent surgical resection from 2012 to 2013 for immunohistochemistry staining and overall survival (OS) and recurrence-free survival (RFS) rate. Freshly resected CRC tissues and adjacent normal tissues were obtained immediately after surgical resection in 2020. Freshly resected CRC tissues and adjacent normal tissues were obtained immediately after surgical resection from two patients with large CRC for single-cell RNA-seq analysis. Two patients have muscularis propria invasive CRC without lymph node or distant metastasis, and subserosal invasive CRC with liver metastasis.

This study was approved by the Research Ethics Committee of Osaka University (approval no. 19020). Written informed consent was obtained from all patients.

### Flow cytometry

Normal colon tissues were obtained from the intact surrounding tissues. Cells were immediately isolated after collection. The intestinal mucosa was washed with phosphate-buffered saline (PBS), cut into small pieces, and incubated in Roswell Park Memorial Institute (RPMI) 1640 containing 10% foetal bovine serum (FBS), 2 mg/mL of collagenase D (Roche, Basel, Switzerland) and 15 μg/mL of DNase I (Roche) for 60 min in a shaking water bath at 37 °C to isolate normal colonic mucosa cells. The digested tissues were passed through a 40-μm cell strainer. Next, the isolated cells were washed with RPMI 1640, incubated in ACK buffer (3 mL, incubating for 3 min to lyse red blood cells), and washed again with RPMI 1640. Normal colonic mucosa cells were collected in PBS containing 2% FBS. Isolated cells were stained with surface antibodies for 30 min at 4 °C, followed by 7AAD staining (BD Biosciences, Franklin Lakes, NJ). Flow cytometric analysis and cell sorting were performed using FACSAria II (BD Biosciences). Data were analysed using FlowJo software (Tree Star, San Carlos, CA).

### Immunohistochemical staining

Immunohistochemistry staining was performed on formalin-fixed, paraffin-embedded tissue sections (4.0 mm). Antigen retrieval was performed using 10 mmol/L of citrate buffer (pH 6) after deparaffinization, and intrinsic peroxidase activity was blocked using 3% H_2_O_2_ for 20 min, followed by nonspecific interaction blocking with Background Sniper (Biocare Medical, Pacheco, CA) for 10 min. Anti-CD103 (ab129202, rabbit, diluted 1:2000) and anti-CD8 (ab75129, mouse, diluted 1:50) antibodies were used to stain tissue sections using the Vectastain ABC kit (Vector Laboratories, Burlingame, CA). The number of CD103^+^ T cells and CD8^+^ T cells was counted in the tumour-invasive margins using ImageJ software version 1.8.0 (NIH, Bethesda, MD, USA; http://imagej.nih.gov/ij).

### Single-cell RNA-seq analyses

The same method as flow cytometry was used to obtain cells by isolation and staining with CD45 antibody (#480029 MojoSort™ Human CD45 Nanobeads) to isolate CD45^+^ cells using only MACS. The BD Rhapsody Single-Cell Analysis System (BD Biosciences) was used to target CD45^+^ cells for single-cell RNA-seq analysis. Briefly, the single-cell suspension was loaded into a BD Rhapsody cartridge with >200,000 microwells, and single-cell capture was achieved by random distribution and gravity precipitation. Then, the bead library was loaded into the microwell cartridge to saturation so that each bead was paired with a cell in a microwell. The cells were lysed in a microwell cartridge to hybridise mRNA molecules onto bar-coded capture oligos on the beads. Then, these beads were retrieved from the microwell cartridge into a single tube for subsequent complementary DNA (cDNA) synthesis, exonuclease I digestion, and multiplex–polymerase chain reaction (PCR)-based library construction. The Illumina library was converted to a library for DNBSEQ using the MGIEasy Universal Library Conversion Kit (App-A). Sequencing was performed on the DNBSEQ-G400RS (MGI) in the 100-base paired-end mode. The BD Rhapsody Analysis Pipeline was used to process sequencing data (fastq files), and output result files were analysed and visualised using BD Data View software v.1.2.2 (BD Biosciences). CD45^+^ cells were clustered by t-SNE analysis, and comparisons were made between clusters.

The GSE108989, GSE146711, and GSE164522 single-cell RNA-seq data were downloaded in Gene Expression Omnibus (https://www.ncbi.nlm.nih.gov/geo/).

The R package Seurat version 3.1.5 was used to analyse GSE108989, GSE146771, and GSE164522 gene sets. The cited gene expression matrixes from GSE108989 were read into R version 4.0.1 and converted to Seurat objects. The subsequent analysis only included tumour-infiltrating T cells. Principal component analysis was performed based on highly variable genes after scaling the data concerning unique molecular identifier counts to reduce dimensionality. Principle components were selected for downstream clustering based on the heatmap, jackstraw plot, and elbow plot of principal components to further reduce dimensionality using the t-SNE algorithm. We corrected batch effects as follows when simultaneously analysing tissue from two patients.

We used Seurat for single-cell RNA-seq integration. Seurat includes a set of methods to match shared cell populations across datasets. These methods first identify cross-dataset pairs of cells that are in a matched biological state (“anchors”), can be used both to correct for technical differences between datasets (batch effect correction), and to compare single-cell RNA-seq analysis between experimental conditions. We normalise and identify variable features for each dataset independently, and select features that are variable across datasets for integration. We then identify anchors using the FindIntegrationAnchors function, which takes a list of Seurat objects as input and use these anchors to integrate the two datasets together with IntegrateData.

### Statistical analysis

The Wilcoxon and Fisher’s exact probability tests were used to determine statistically significant differences between groups. The Kaplan–Meier method and the log-rank test were used to calculate OS and RFS rates. All statistical analyses were performed using R programming language version 4.0.2 and JMPpro 14.0.0 (SAS Institute, Cary, NC, USA). *P* values of <0.05 were considered statistically significant.

## Results

### CD103, a Trm cell marker, is associated with cytotoxic T-cell activation and is a prognostic predictive factor in CRC

**Fig. 1:**
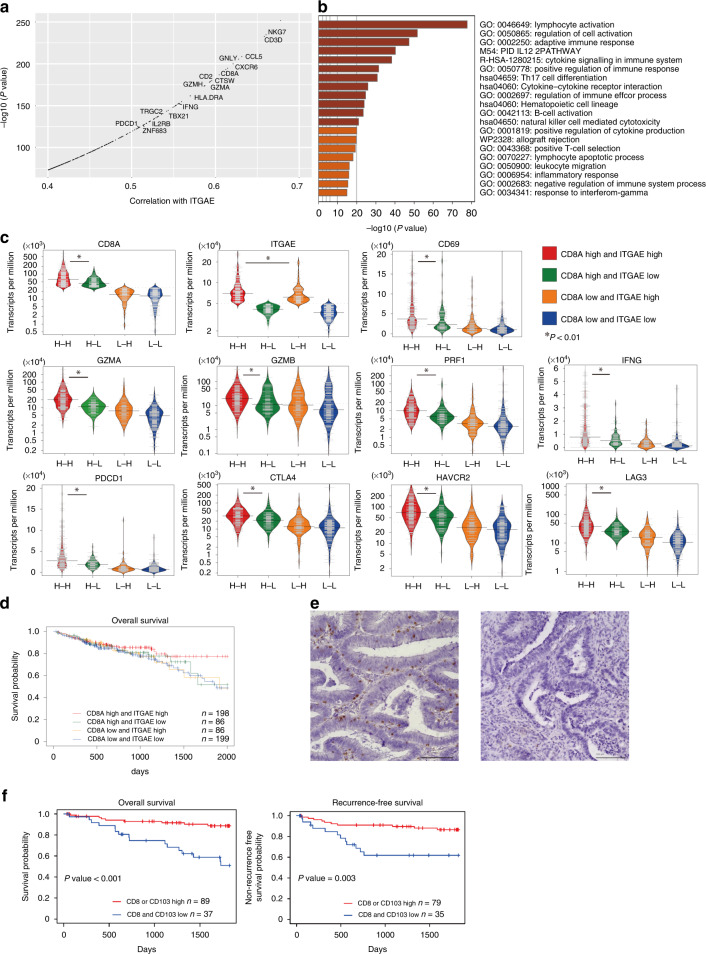
CD103, which drives Trm cell generation, correlates with lymphatic activation in CRC. **a** ITGAE-correlated genes encoding CD103 in TCGA-COAD and TCGA-READ. **b** Bar graph showing enriched terms across the gene sets correlated with ITGAE (correlation coefficient >0.4), coloured by *P* values. **c** Bean plots of cytotoxic cytokines, immune checkpoint molecules, and Trm cells are considered core genes of CRC in TCGA. The genes were categorised according to the median mRNA expression of CD8A and ITGAE as cut-off values into high CD8A and high ITGAE (H–H), high CD8A and low ITGAE (H–L), low CD8A and high ITGAE (L–H), and low CD8A and low ITGAE (L–L) groups. **d** Kaplan–Meier curve of OS for the four groups. **e** Immunohistochemical staining for CD103 at invasive margins of resected CRC tissue (left: high invasion; right: low invasion). **f** Kaplan–Meier curves of OS and RFS by CD103^+^ and CD8^+^ cell count. Trm cells tissue-resident memory T cells, CRC colorectal cancer, ITGAE integrin subunit alpha E, TCGA *The Cancer Genome Atlas,* COAD colon adenocarcinoma, READ rectum adenocarcinoma, mRNA messenger RNA, OS overall survival, RFS recurrence-free survival.

Trm cell subsets from resected CRC tissue were identified via flow cytometry to identify a tumour-specific Trm cell marker and to determine whether Trm cell infiltration could be a prognostic factor in CRC. CD8^+^ T cells expressing the Trm cell marker CD103 were almost always positive for CD69, which is also a recognised Trm cell marker (Supplementary Fig. 1). Thus, Trm cells were defined as CD8^+^ and CD103^+^ T cells. RNA sequencing (RNA-seq) data of 569 CRC cases with prognostic data (420 colon adenocarcinoma [COAD] and 149 rectal adenocarcinoma [READ] cases) were downloaded from *The Cancer Genome Atlas* (TCGA; http://tcga-data.nci.nih.gov) data portal, and gene expression levels that correlated with the CD103 level were analysed to investigate the role of CD103 in CRC. Integrin subunit alpha E (ITGAE; encoding CD103) was strongly correlated with the expression of genes associated with cytotoxic T cells, such as *CD8A*, *GZMA* and *IFNG*, in both TCGA-COAD and TCGA-READ (Fig. 1a). Metascape analysis revealed that genes with correlation coefficients of >0.4 for ITGAE were involved in lymphocyte activation (Fig. 1b). Cases were classified into four groups according to the median messenger RNA (mRNA) expression of CD8A and ITGAE as cut-off values: high CD8A and high ITGAE (H–H), high CD8A and low ITGAE (H–L), low CD8A and high ITGAE (L–H), and low CD8A and low ITGAE (L–L). The H–H group had high cytotoxic cytokines, immune checkpoint molecules, and Trm-related gene expression (Fig. 1c). Prognostic analysis was further performed using these data. Kaplan–Meier survival curves revealed that the H–H group had a better OS than the H–L group (Fig. 1d). We observed a trend toward a longer OS in the H–H and L–H groups by comparing the OS with ITGAE expression alone using other CRC RNA sequence datasets (GSE41258, GSE28814 and TCGA) (Supplementary Fig. 2). Surgically resected specimens from 126 patients with CRC were immunohistochemically stained with CD103 and CD8 antibodies to detect CD103^+^/CD8^+^ lymphocyte infiltration (Fig. 1e, f, Supplementary Fig. 3 and Supplementary Tables 1 and 2). The number of CD103^+^/CD8^+^ tumour-infiltrating lymphocytes (TILs) was a favourable prognostic and predictive factor of the OS and RFS (Fig. 1g, *P* < 0.05). Univariate and multivariate analyses revealed that a low number of CD8^+^ and CD103^+^ TILs is an independent poor prognostic factor of OS and RFS (Table 1 and Supplementary Table 3). The number of CD103^+^ TILs alone is a prognostic predictive factor of OS and RFS and was significantly more associated with pathological factors (lymphatic invasion, lymph node metastasis and distant metastasis) compared with the number of CD8^+^ TILs (Supplementary Figs. 4 and 5). These results suggest that the Trm cell markers CD8 and CD103 positive TILs are prognostic factors in patients with CRC.

**Table 1:** Factors evaluated for OS (univariate and multivariate analyses).

OS	Univariate analysis			Multivariate analysis		
Variables	HR	95% CI	*P* value	HR	95% CI	*P* value
Age (>=65 years)	1.93	0.87–4.31	0.098			
Sex (male)	1.11	0.51–2.43	0.792			
BMI (<25 kg/m²)	1.56	0.73–3.38	0.260			
Tumour location (rectum)	1.03	0.48–2.20	0.937			
Histological type (undifferentiated)	2.34	0.88–6.21	0.089			
Greatest tumour diameter (≧35 mm)	3.24	1.30–8.03	0.006	1.70	0.50–5.71	0.389
Pathological T category (T2–T4)	4.12	0.96–17.6	0.056			
Lymphatic invasion (+)	2.02	0.91–4.50	0.086			
Vascular invasion (+)	2.98	1.36–6.55	0.007	1.71	0.71–4.16	0.234
Lymph node metastasis (+)	1.75	0.81–3.78	0.154			
Distant metastasis (+)	5.58	2.49–12.5	<0.001			
Pathological Stage (III–IV)	3.56	1.43–8.83	0.006	2.12	0.61–7.48	0.239
LL (CD103^+^ < 60 /10HPF and CD8^+^ < 62/10HPF)	3.48	1.38–8.78	0.001	2.81	1.08–7.31	0.034

*OS* overall survival, *HR* hazard ratio, *CI* confidence interval, *BMI* body mass index, *LL* the numbers of both CD103^+^ and CD8^+^ cells were low, *HPF* high-power field.

### Trm cells in CRC are the activated population of CD8^+^ T cells

**Fig. 2:**
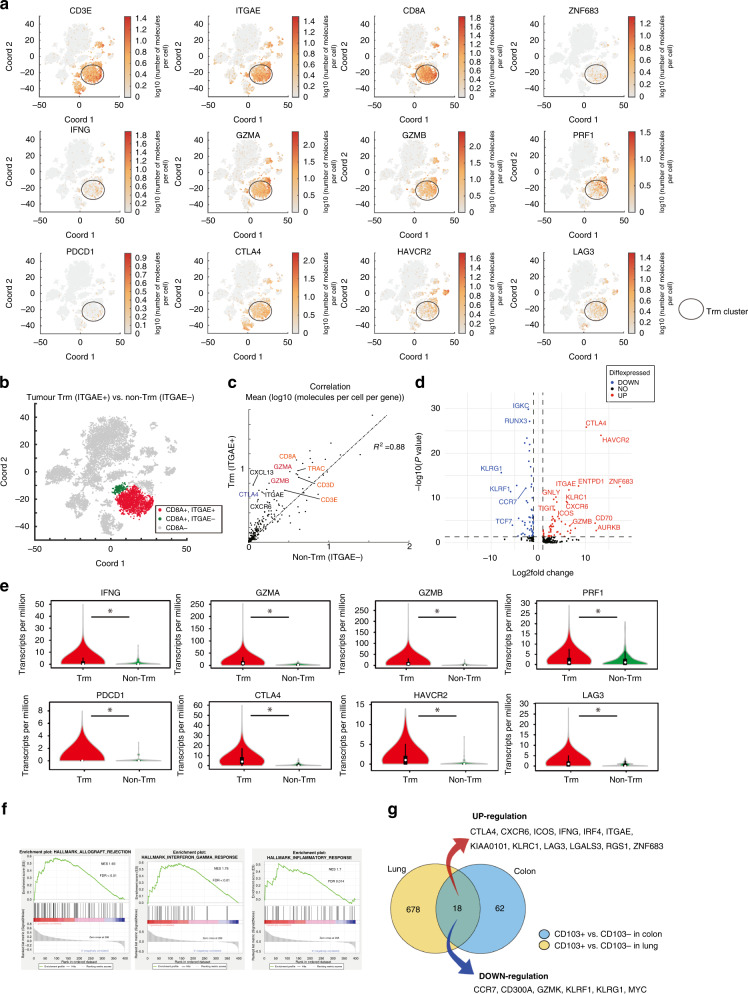
Trm cells represent the most activated subset of CD8^+^ T cells in CRC. **a** t-SNE plots of ~12,000 live and singlet-gated CD45^+^ single-cell transcriptomes obtained from resected CRC tissue showing gene expression encoding Trm cell markers, cytotoxic cytokines, and immune checkpoint molecules. **b** Trm (red) and non-Trm (green) clusters are defined according to ITGAE (encoding CD103) and CD8A expression. **c** Correlation between Trm and non-Trm cell clusters: mean (log_10_ [molecules/cell/gene]). **d** Volcano plot showing DEGs between Trm and non-Trm cell clusters. Dot lines represent an FDR of <0.05 and a fold change of >2. **e** Violin plot of the expression of cytotoxic cytokines and immune checkpoint molecule genes in the two clusters in (**b**). **f** Top 3 NES of hallmark gene sets in GSEA between Trm and non-Trm cell clusters. **g** Venn diagrams overlapping transcripts differentially expressed with a fold change of >2 between Trm and non-Trm cell clusters in lung cancer (left) and CRC (right). Trm cells tissue-resident memory T cells, CRC colorectal cancer, ITGAE integrin subunit alpha E, t-SNE t-distributed stochastic neighbour embedding, DEG differentially expressed gene, FDR false discovery rate, NES normalised-enrichment scores, GSEA gene set enrichment analysis.

The number of Trm cells is correlated with the pathological depth of invasion and lymph node and distant metastasis, OS, and RFS of CRCs. Thus, we investigated the characteristics of CRC Trm cells. Intratumor 17257 immune cells were obtained from resected CRC tissues of two patients, and single-cell RNA-seq analysis was performed to investigate the characteristics of Trm cells. The t-distributed stochastic neighbour embedding (t-SNE) plot showed that ITGAE^+^ cells co-express CD3D or CD3E and commonly co-express CD8A. Cells co-expressing ITGAE and CD8A were identified as Trm cells, and Trm cell clusters revealed a higher gene expression of cytotoxic cytokines and immune checkpoint molecules (Fig. 2a). ITGAE^+^ CD8A^+^ T-cell and ITGAE^–^CD8A^+^ T-cell clusters were defined as Trm cell (red area) and non-Trm cell (green area) clusters to compare Trm and non-Trm cells, respectively (Fig. 2b). The Trm cell cluster had a higher gene expression of cytotoxic cytokines and immune checkpoint molecules compared with the non-Trm cell cluster. The expression of the gene related to cytotoxic T cells in the Trm cell cluster was stronger compared with the non-Trm cell cluster although the Trm cell cluster showed a strong correlation with the non-Trm cell cluster (Fig. 2c). Expression of the exhaustion markers CTLA4, HAVCR2 and ENTPD1 were greater in the Trm cell population than in the non-Trm cell cluster (Fig. 2d). Gene expression of cytotoxic cytokines and immune checkpoint molecules was also significantly higher in the Trm cell cluster (Fig. 2e). The top-ranked genes were gene sets related to the inflammatory response and *IFNG* response in gene set enrichment analysis (GSEA) (Fig. 2f). In addition, we searched for gene expression in tumour-infiltrating Trm by comparing CD8^+^ CD103^+^ T cells with CD8^+^ CD103^−^ T cells in lung cancer in GSE111898, which is a dataset comprising the results a single-cell analysis of lung cancer. We compared the genes that showed a fold change of >2 in Trm cells from lung cancer with genes that revealed a fold change of >2 in Trm cells from colon cancer. Similar movements were observed in 18 genes in Trm cells in lung cancer, and these genes may be central in Trm cells (Fig. 2g).

Trm cells in CRC were a particularly cytotoxic cell population among CD8^+^ T cells. In addition, Trm cells showed high expression of immune checkpoint molecules, termed exhaustion markers.

### High-infiltrating Trm cells in cancer highly expressed ZNE683

**Fig. 3:**
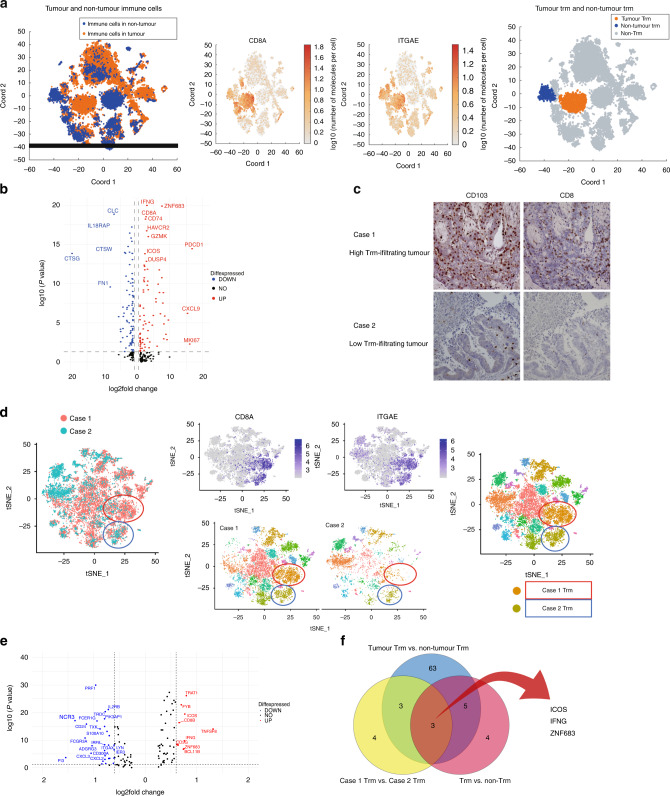
Characteristics of cancer-specific Trm cells in CRC. **a** t-SNE plots of singlet-gated CD45^+^ single-cell transcriptomes obtained from resected CRC and adjacent normal colon tissue and gene expression of ITGAE and CD8A. Tumour Trm (orange) and non-Trm (blue) cell clusters are defined according to ITGAE and CD8A expression. **b** Volcano plot showing the DEGs between Trm and non-Trm cell clusters. **c** Immunohistochemical staining of CRC tissues at the invasive margins; CD103 staining of Case 1 (high invasion; upper left), CD8 staining of Case 1 (high invasion; upper right), CD103 staining of Case 2 (low invasion; lower left) and CD8 staining of Case 2 (low invasion; lower right). **d** t-SNE plots of singlet-gated CD45^+^ single-cell transcriptomes obtained from Trm high- and low invasion cases of resected CRC. High (orange) and low (dark yellow) Trm invasion are defined according to ITGAE and CD8A expression. **e** Volcano plot showing DEGs between Case 1 and Case-2 Trm cell clusters. **f** Venn diagram of upregulated genes in Fig. 2e, tumour Trm cells, and high invasion Trm cells. Trm cells tissue-resident memory T cells, CRC colorectal cancer, ITGAE integrin subunit alpha E, t-SNE t-distributed stochastic neighbour embedding, DEG differentially expressed gene.

We compared cancer Trm cells with noncancer Trm cells to identify the characteristics of cancer Trm cells. Figure 3a shows t-SNE plots representing immune cells in cancer and noncancer areas, with cancer Trm cell (orange area) and noncancer Trm cell (blue area) clusters defined. Volcano plot showing differentially expressed genes (DEGs) between Trm and non-Trm cell clusters revealed that Trm clusters were more characterised by higher gene expression related to inflammation (e.g., GZMK, IFNG, CD8A), immune checkpoint molecules (e.g., PDCD1, HAVCR2, ICOS, DUSP4), and proliferative potential (MKi67) (Fig. 3b). We compared the gene expression of Trm cells between cases with high and low Trm cell infiltration to identify the characteristic of cancer-specific Trm cells. We assumed that Trm cells in this group recognise the tumour because the OS of patients with CRC was better in the high Trm infiltration group. Single-cell analysis of tumour-infiltrating immune cells from two resected CRC cases was performed and a t-SNE plot was generated to compare Trm cells between high- and low-infiltrating Trm cell cases. Characteristics and immunostaining count data of two cases are shown in Supplementary Table 4. The number of infiltrating Trm cells was higher in Case 1 than in Case 2 in the resected immunostained specimens (Fig. 3c). t-SNE plot showed Case 1 and Case-2 immune cells. Trm cell clusters were defined based on CD8A and ITGAE expression (red circle and blue circle) (Fig. 3d). Trm cluster in red circle consisted of almost Case 1 Trm cells and Trm cluster in blue circle consisted of both of Case 1 and Case-2 Trm. Most Case-2 Trm was in the light green circle. The Trm cluster in the red circle was defined as the high-infiltrating Trm cell cluster. The Trm cluster in the blue circle was defined as the low-infiltrating Trm cell cluster. The high-infiltrating Trm cluster was compared with the low-infiltrating Trm cluster, and the volcano plot revealed highly expressed gene in the high-infiltrating Trm cluster (Fig. 3e). ZNF683 expression was higher in cancer Trm cells than in noncancer Trm cells, and was also upregulated in lung cancer Trm cells (Fig. 3f). ZNF683 was more upregulated in high-infiltrating Trm cells in cancer, which may be related to cancer-specific Trm than low-infiltrating Trm in cancer.

### The characteristics of ZNF683^+^ Trm

**Fig. 4:**
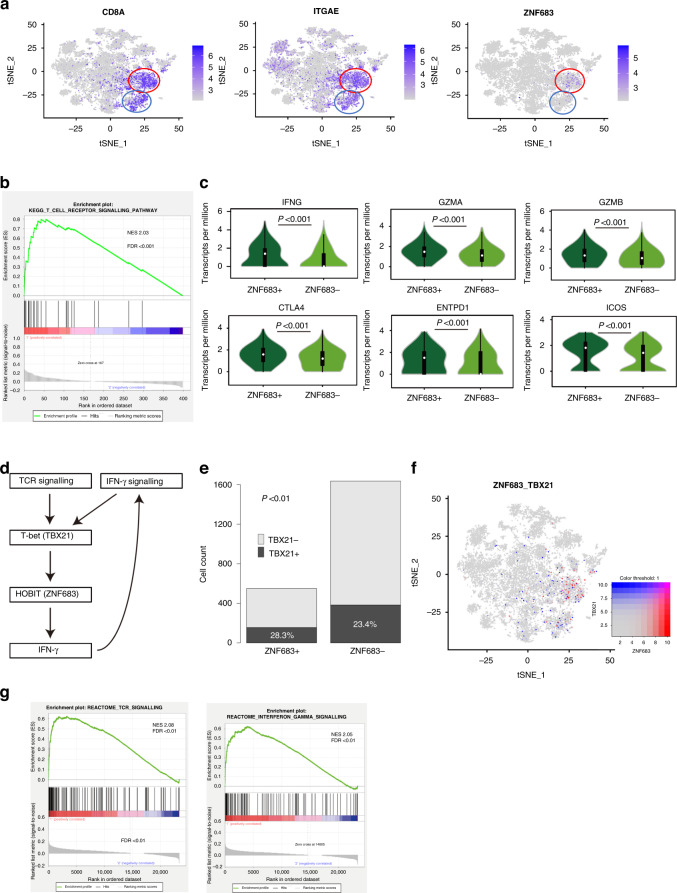
ZNF683^+^ Trm cells enhance tumour suppression through positive feedback by autocrine IFN-γ. **a** Distribution of CD8A +, ITGAE + and ZNF683^+^ cells in the t-SNE plot. **b** GSEA comparing ZNF683^+^ and ZNF683^–^ Trm cell clusters. **c** Violin plot showing the expression of cytotoxic cytokines and immune checkpoint molecules in association with ZNF683 expression. **d** The hypothesis of signals surrounding ZNF683. **e** TBX21–ZNF683 expression ratio in Trm cells. **f** t-SNE plot of cells expressing ZNF683 and TBX21 (red, ZNF683; blue, TBX21). **g** High ITGAE and high-CD8A cases with high expression of ZNF683 in the TCGA database; TOP4 signalling was elevated in GSEA was IFN-γ and TCR signalling. ZNF683 zinc finger protein 683, Trm cells tissue-resident memory T cells, IFN-γ interferon-gamma, t-SNE t-distributed stochastic neighbour embedding, GSEA gene set enrichment analysis, TCGA *The Cancer Genome Atlas*, ITGAE integrin subunit alpha E, TCR T-cell receptor.

*ZNF683*, which is a transcription factor, is a homologous gene of B-lymphocyte-induced maturation protein-1 (*BLIMP-1*) and has been reported as a core gene of Trm cells. However, the single-cell analysis revealed the existence of both Trm cells with and without ZNF683 expression (Fig. 4a). We determined the characteristics of ZNF683^+^ Trm cells by comparing ZNF683^+^ and ZNF683^–^ Trm cells because ZNF683 was hypothesised as a cancer-specific marker. ZNF683^+^ Trm cells revealed increased expression of gene sets related to the T-cell receptor (TCR) signalling pathway in GSEA (Fig. 4b). Moreover, the expression of cytotoxic cytokines and immune checkpoint molecules was higher in ZNF683^+^ than in ZNF683^–^ Trm cells (Fig. 4c). Next, we investigated the pathways associated with ZNF683 expression in Trm cells. We hypothesised a pathway for ZNF683 expression based on previous reports on ZNF683 (Fig. 4d). We searched for the expression of the transcription factor TBX21, which has been reported to exist upstream of ZNF683 [33]. TBX21 expression was higher in ZNF683^+^ than in ZNF683^–^ Trm cells, and the ratio of TBX21 positive cells was higher in ZNF683^+^ than in ZNF683^–^Trm cells (Fig. 4e, f). TBX21 has been reported to be upregulated by stimuli such as TCR signalling and IFN-γ signalling [33, 34], thereby supporting the hypothesis that ZNF683^+^ Trm cells are cancer-specific Trm cells. In addition, IFNG expression was upregulated in ZNF683^+^ Trm cells. Cases with high ZNF683 expression in the TCGA database revealed gene set enrichment of TCR and IFN-γ signalling (Fig. 4g). ZNF683^+^ Trm cells revealed activated TCR signalling. In addition, TCR signalling was shown to upregulate ZNF683 expression via TBX21, and the positive feedback from IFN-γ signalling to ZNF683 is triggered by IFNG upregulation. We analysed data from single-cell analysis of 59364 CRC-infiltrating immune cells in 39 CRC cases (GSE108989, GSE146711 and GSE164522) to confirm the accuracy of single-cell analysis results. The Trm cell cluster revealed high expression of cytotoxic cytokines (Supplementary Fig. 6a). CD8A^+^ and ITGAE^+^ cells, which are considered Trm cells, were divided into two groups according to the presence or absence of ZNF683 expression. The ZNF683^+^ Trm cell cluster revealed significantly higher TBX21 and IFNG expression (Supplementary Fig. 6b). GSEA revealed that ZNF683^+^ Trm cells showed significantly higher TCR and IFN-γ signalling compared with ZNF683^–^ Trm cells (Supplementary Fig. 6c). The ZNF683^+^ Trm cell population showed gene set enrichment of TCR and IFN-γ signalling in addition to our dataset. Moreover, the TBX21 and IFNG expression in ZNF683^+^ Trm cells was upregulated.

## Discussion

This study determined the characteristics of cancer-specific Trm cells. We identified ZNF683 as one of the makers of cancer-specific Trm cells, which appear to have high cytotoxicity, by comparing Trm cells under various conditions at the single-cell level. We hypothesised that ZNF683 expression is possibly mediated by self-positive feedback via IFN-γ signalling.

Trm cells can induce a potent antitumor immune response in various solid tumours [12]. However, the functions and prognostic significance of Trm cells in CRC have not been systematically addressed. The CD8^+^ T-cell infiltration level in CRC is an independent prognostic factor [35–37]. Trm cells are a useful predictive factor of prognosis in CRC in this study, and the upregulated gene of the high-infiltrating Trm cluster was ZNF683 in single-cell analysis. This study revealed ZNF683 as a marker for cancer-specific Trm, assuming that tumours with high Trm infiltration have a high number of cancer-specific Trm cells. ZNF683^+^ Trm cells revealed higher expression of cytotoxic cytokines and immune checkpoint molecules and activated TCR signalling, which suggests that ZNF683 + Trm plays an antitumor role within the tumour. Moreover, ZNF683^+^ Trm cells revealed high expression of TBX21, which acts upstream of ZNF683 [2, 28].

TBX21 has various signals upstream, one of which is TCR stimulation [33, 34]. IFN-γ signalling was reported as an upstream signal of TBX21 [33, 38, 39]. In this study, IFNG expression was upregulated in ZNF683^+^ Trm cells, suggesting the feasibility of IFN-γ autocrine positive feedback [40]. IFN-γ is a cytokine with high antitumor effects, and ZNF683^+^ Trm cells upregulate IFNG expression and enhance its antitumor effect.

This study has some limitations. First, we were unable to confirm whether ZNF683^+^ Trm cells could recognise or attack tumours because our analysis was performed only at the gene expression level in cells. However, a recent study suggested that ZNF683 is upregulated in neoantigen-specific TILs in lung cancer [41], which was consistent with our findings. Second, we were unable to confirm whether ZNF683^+^ Trm cells could affect long-term prognosis, including OS, in CRC because we used freshly resected specimens. However, the presence of cancer-specific Trm cells is likely to positively impact tumour prognosis because Trm cells are associated with tumour prognosis. Third, we were unable to conduct a detailed analysis of the relationship among ZNF683, TBX21, and IFNG. Additionally, the mechanism by which TBX21 upregulates ZNF683 expression and the effect of ZNF683 on increased IFNG expression were not verified. However, previous studies have reported that TBX21 is required for ZNF683 expression and that TBX21 is a feedback factor in IFN-γ signalling [2, 28, 42]. Hence, ZNF683 is assumed as feedback to the IFNG that it produces, leading to increased IFNG expression.

This study revealed that only a portion of Trm cells in the tumour is activated, suggesting the existence of cancer-specific and non-cancer-specific Trm cell populations. The passage of food residues and the development of bacterial flora in the colorectum results in the presence of many antigens; thus, we can assume various Trm cells specific to certain antigens. Therefore, identifying cancer-specific Trm cells is important. ZNF683^+^ Trm cells, identified as cancer-specific Trm cells in this study, have higher IFNG and GZMB expression compared with ZNF683^–^ Trm cells, suggesting that cancer-specific Trm cells have high cytotoxic potential. This suggests that cancer-specific Trm cells would have exceptionally high tumour immunity among Trm cells.

*ZNF683*, also known as Hobit, is a transcription factor that is a homologous gene of *BLIMP-1* [43]. The single-cell analysis revealed the existence of ZNF683^+^ and ZNF683^–^Trm cell subsets although increased *ZNF683* expression in Trm cell populations indicates that *ZNF683* is a core gene [27]. The ZNF683 expression is not well understood; however, our results suggest that ZNF683 expression is activated by TCR or IFN-γ signalling stimulation. Reports on genes upstream of ZNF683 are limited, and *TBX21* is one of the few upstream genes reported [33–37]. TBX21 encodes the protein T-bet, which is a positive IFN-γ regulator, the signature cytokine of Th1 cells [44, 45]. However, this study expressed TBX21 in Trm cells, which not only play a role in initial tumour immunity but also have a strong influence on other immune cells, such as Th1 cells, due to IFN-γ expression. ZNF683^+^ Trm cells play a central role in cellular immunity, especially tumour immunity.

In conclusion, the number of tumour-infiltrating CD103^+^ Trm cells is a prognostic predictive factor in the OS and RFS in CRC, and tumour-infiltrating CD103^+^ Trm cells predominantly comprise CD8 T cells expressing cytotoxic cytokines and immune checkpoint molecules. Additionally, we identified cancer-specific Trm cells as cancer-specific Trm cells. ZNF683^+^ Trm cells could play a role in tumour immunity by expressing IFN-γ (Supplementary Fig. 6d).

## Supplementary information

SUPPLEMENTAL MATERIAL

authorship

## Data Availability

The processed gene expression data were deposited in the Gene Expression Omnibus database under accession id GSE 188381.

## References

[1] Medzhitov R, Janeway CA (2002). Decoding the patterns of self and nonself by the innate immune system. Science.

[2] Ehrlich P (1909). Über den jetzigen Stand der Karzinomforschung. Ned Tijdschr Geneeskd..

[3] Amsen D, van Gisbergen KPJM, Hombrink P, van Lier RAW (2018). Tissue-resident memory T cells at the center of immunity to solid tumors. Nat Immunol..

[4] Webb JR, Milne K, Watson P, DeLeeuw RJ, Nelson BH (2014). Tumor-infiltrating lymphocytes expressing the tissue resident memory marker CD103 are associated with increased survival in high-grade serous ovarian cancer. Clin Cancer Res.

[5] Sheridan BS, Lefrançois L (2011). Regional and mucosal memory T cells. Nat Immunol..

[6] Sallusto F, Lenig D, Förster R, Lipp M, Lanzavecchia A (1999). Two subsets of memory T lymphocytes with distinct homing potentials and effector functions. Nature.

[7] Schenkel JM, Masopust D (2014). Tissue-resident memory T cells. Immunity.

[8] Schön MP, Arya A, Murphy EA, Adams CM, Strauch UG, Agace WW (1999). Mucosal T lymphocyte numbers are selectively reduced in integrin αE (CD103)-deficient mice. J Immunol.

[9] Machado-Santos J, Saji E, Tröscher AR, Paunovic M, Liblau R, Gabriely G (2018). The compartmentalized inflammatory response in the multiple sclerosis brain is composed of tissue-resident CD8+ T lymphocytes and B cells. Brain.

[10] Kuric E, Seiron P, Krogvold L, Edwin B, Buanes T, Hanssen KF (2017). Demonstration of tissue resident memory CD8 T cells in insulitic lesions in adult patients with recent-onset type 1 diabetes. Am J Pathol..

[11] Zundler S, Becker E, Spocinska M, Slawik M, Parga-Vidal L, Stark R (2019). Hobit-and Blimp-1-driven CD4+ tissue-resident memory T cells control chronic intestinal inflammation. Nat Immunol..

[12] Sasson SC, Gordon CL, Christo SN, Klenerman P, Mackay LK (2020). Local heroes or villains: tissue-resident memory T cells in human health and disease. Cell Mol Immunol..

[13] Park SL, Buzzai A, Rautela J, Hor JL, Hochheiser K, Effern M (2019). Tissue-resident memory CD8^+^ T cells promote melanoma-immune equilibrium in skin. Nature.

[14] Wang ZQ, Milne K, Derocher H, Webb JR, Nelson BH, Watson PH (2016). CD103 and intratumoral immune response in breast cancer. Clin Cancer Res.

[15] Djenidi F, Adam J, Goubar A, Durgeau A, Meurice G, de Montpréville V (2015). CD8+ CD103+ tumor–infiltrating lymphocytes are tumor-specific tissue-resident memory T cells and a prognostic factor for survival in lung cancer patients. J Immunol.

[16] Komdeur FL, Prins TM, van de Wall S, Plat A, Wisman GBA, Hollema H (2017). CD103+ tumor-infiltrating lymphocytes are tumor-reactive intraepithelial CD8+ T cells associated with prognostic benefit and therapy response in cervical cancer. Oncoimmunology.

[17] Masopust D, Vezys V, Marzo AL, Lefrançois L (2001). Preferential localization of effector memory cells in nonlymphoid tissue. Science.

[18] Badoual C, Hans S, Rodriguez J, Peyrard S, Klein C, Agueznay Nel H (2006). Prognostic value of tumor-infiltrating CD4+ T-cell subpopulations in head and neck cancers. Clin Cancer Res.

[19] Badoual C, Hans S, Merillon N, Van Ryswick C, Ravel P, Benhamouda N (2013). PD-1–expressing tumor-infiltrating T cells are a favorable prognostic biomarker in HPV-associated head and neck cancer. Cancer Res.

[20] Welters MJP, Ma W, Santegoets SJAM, Goedemans R, Ehsan I, Jordanova ES (2018). Intratumoral HPV16-specific T cells constitute a type I–oriented tumor microenvironment to improve survival in HPV16-driven oropharyngeal cancer. Clin Cancer Res.

[21] Duhen T, Duhen R, Montler R, Moses J, Moudgil T, de Miranda NF (2018). Co-expression of CD39 and CD103 identifies tumor-reactive CD8 T cells in human solid tumors. Nat Commun..

[22] Savas P, Virassamy B, Ye C, Salim A, Mintoff CP, Caramia F (2018). Single-cell profiling of breast cancer T cells reveals a tissue-resident memory subset associated with improved prognosis. Nat Med..

[23] Ganesan AP, Clarke J, Wood O, Garrido-Martin EM, Chee SJ, Mellows T (2017). Tissue-resident memory features are linked to the magnitude of cytotoxic T cell responses in human lung cancer. Nat Immunol..

[24] Wang B, Wu S, Zeng H, Liu Z, Dong W, He W (2015). CD103+ tumor infiltrating lymphocytes predict a favorable prognosis in urothelial cell carcinoma of the bladder. J Urol..

[25] Hartana CA, Ahlén Bergman E, Broomé A, Berglund S, Johansson M, Alamdari F (2018). Tissue‐resident memory T cells are epigenetically cytotoxic with signs of exhaustion in human urinary bladder cancer. Clin Exp Immunol..

[26] Edwards J, Wilmott JS, Madore J, Gide TN, Quek C, Tasker A (2018). CD103+ tumor-resident CD8+ T cells are associated with improved survival in immunotherapy-naïve melanoma patients and expand significantly during anti-PD-1 treatment. Clin Cancer Res.

[27] Kurd NS, He Z, Louis TL, Milner JJ, Omilusik KD, Jin W, et al. Early precursors and molecular determinants of tissue-resident memory CD8+ T lymphocytes revealed by single-cell RNA sequencing. Sci. Immunol. 2020;5:eaaz6894. 10.1126/sciimmunol.aaz6894 PMC7341730 32414833

[28] Mackay LK, Minnich M, Kragten NA, Liao Y, Nota B, Seillet C (2016). Hobit and Blimp1 instruct a universal transcriptional program of tissue residency in lymphocytes. Science.

[29] Cheuk S, Schlums H, Gallais Sérézal I, Martini E, Chiang SC, Marquardt N (2017). CD49a expression defines tissue-resident CD8+ T cells poised for cytotoxic function in human skin. Immunity.

[30] Ariotti S, Hogenbirk MA, Dijkgraaf FE, Visser LL, Hoekstra ME, Song JY (2014). Skin-resident memory CD8+ T cells trigger a state of tissue-wide pathogen alert. Science.

[31] Schenkel JM, Fraser KA, Beura LK, Pauken KE, Vezys V, Masopust D (2014). Resident memory CD8 T cells trigger protective innate and adaptive immune responses. Science.

[32] Yang R, Cheng S, Luo N, Gao R, Yu K, Kang B (2020). Distinct epigenetic features of tumor-reactive CD8+ T cells in colorectal cancer patients revealed by genome-wide DNA methylation analysis. Genome Biol.

[33] Vieira Braga FA, Hertoghs KM, Kragten NA, Doody GM, Barnes NA, Remmerswaal EB (2015). Blimp‐1 homolog Hobit identifies effector‐type lymphocytes in humans. Eur J Immunol..

[34] Pritchard GH, Kedl RM, Hunter CA (2019). The evolving role of T-bet in resistance to infection. Nat Rev Immunol..

[35] Deschoolmeester V, Baay M, Mark EV, Weyler J, Vermeulen P, Lardon F (2010). tumor infiltrating lymphocytes: an intriguing player in the survival of colorectal cancer patients. BMC Immunol.

[36] Naito Y, Saito K, Shiiba K, Ohuchi A, Saigenji K, Nagura H (1998). CD8+ T cells infiltrated within cancer cell nests as a prognostic factor in human colorectal cancer. Cancer Res.

[37] Chiba T, Ohtani H, Mizoi T, Naito Y, Sato E, Nagura H (2004). Intraepithelial CD8+ T-cell-count becomes a prognostic factor after a longer follow-up period in human colorectal carcinoma: possible association with suppression of micrometastasis. Br J Cancer.

[38] Yin Z, Chen C, Szabo SJ, Glimcher LH, Ray A, Craft J (2002). T-Bet expression and failure of GATA-3 cross-regulation lead to default production of IFN-γ by γδ T cells. J Immunol.

[39] Schulz EG, Mariani L, Radbruch A, Hofer T (2002). Sequential polarization and imprinting of type 1-T helper lymphocytes by interferon-γ and interleukin-12. Immunity.

[40] Lugo-Villarino G, Maldonado-Lopez R, Possemato R, Penaranda C, Glimcher LH (2003). T-bet is required for optimal production of IFN-γ and antigen-specific T cell activation by dendritic cells. Proc Natl Acad Sci USA..

[41] Caushi JX, Zhang J, Ji Z, Vaghasia A, Zhang B, Hsiue EH (2021). Transcriptional programs of neoantigen-specific TIL in anti-PD-1-treated lung cancers. Nature.

[42] Iwata S, Mikami Y, Sun H-W, Brooks SR, Jankovic D, Hirahara K (2007). The transcription factor T-bet limits amplification of type I IFN transcriptome and circuitry in T helper 1 cells. Immunity.

[43] Van Gisbergen KP, Kragten NA, Hertoghs KM, Wensveen FM, Jonjic S, Hamann J (2012). Mouse Hobit is a homolog of the transcriptional repressor Blimp-1 that regulates NKT cell effector differentiation. Nat Immunol..

[44] Szabo SJ, Kim ST, Costa GL, Zhang X, Fathman CG, Glimcher LH (2000). A novel transcription factor, T-bet, directs Th1 lineage commitment. Cell.

[45] Mullen AC, High FA, Hutchins AS, Lee HW, Villarino AV, Livingston DM (2001). Role of T-bet in commitment of TH1 cells before IL-12-dependent selection. Science.

