# Supplementary material for: Cancer-specific tissue-resident memory T-cells express ZNF683 in colorectal cancer
Source: Br J Cancer. 2023 Mar 3;128(10):1828–37. doi: 10.1038/s41416-023-02202-4 (PMC10147592; doi:10.1038/s41416-023-02202-4)
Supplement: Supplementary file 1 — SUPPLEMENTAL MATERIAL [file 41416_2023_2202_MOESM1_ESM.pdf]

Supplementary Fig. 1. Selection of Trm markers in colorectal cancer

a. Analysis of the distribution of CD8, CD103, and CD69 expression on T cells ($CD45^+$, $CD3^+$) in the immune cell populations of colorectal cancer tumors using flow cytometry. **b.** The percentage of CD103 expression on $CD8^+$ T cells was approximately 50% of $CD8^+$ T cells, and the percentage varied among cases. **c.** The expression of CD69 in $CD8^+$ $CD103^+$ T cells was more than 90%.

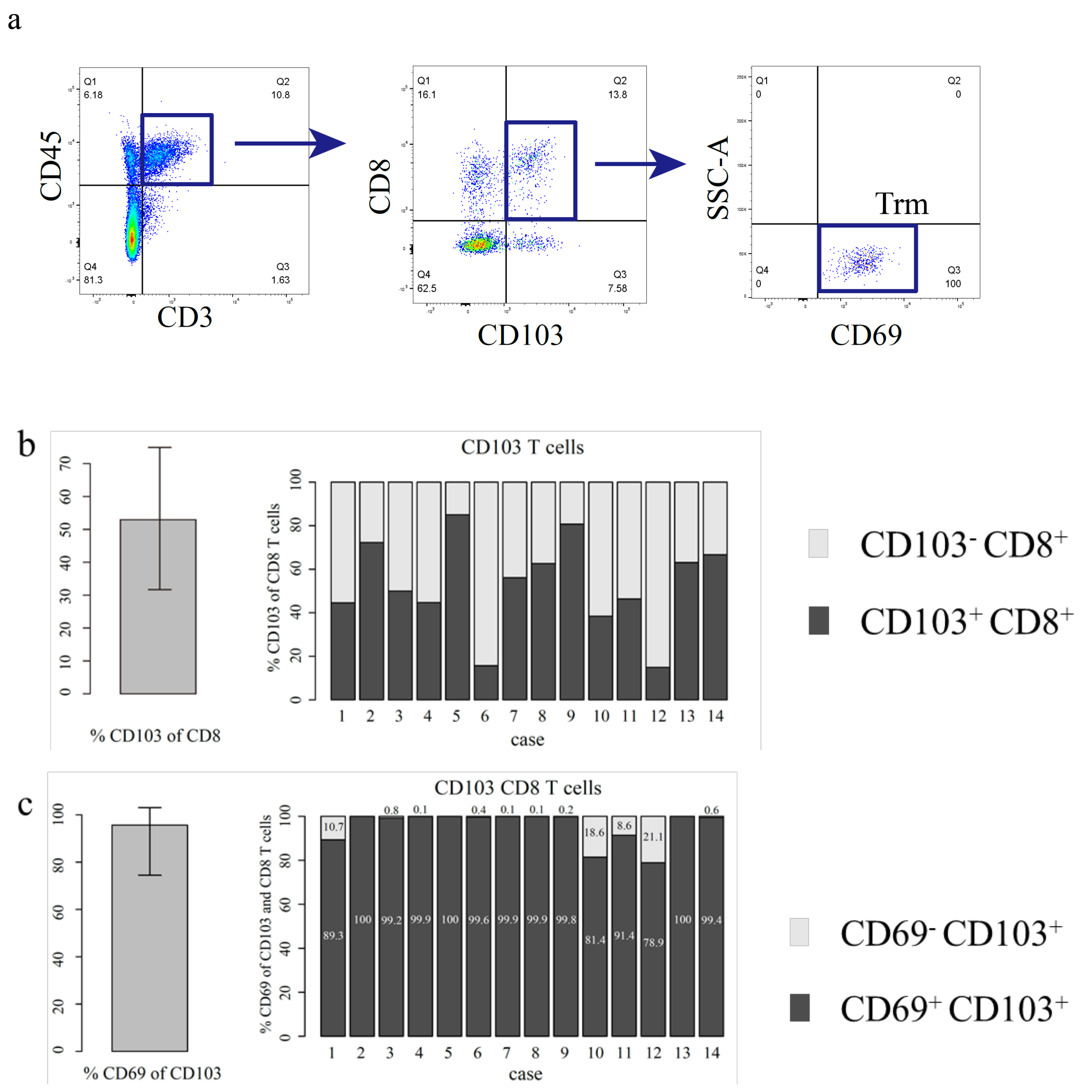

Supplementary Fig. 2. Overall survival by expression of ITGAE in colorectal cancer tissues in each database

The median value of ITGAE expression was used to divide patients into two groups for comparison. In all databases, overall survival tended to be prolonged in patients with higher ITGAE, while in the TCGA database, overall survival was significantly prolonged in patients with higher ITGAE.

a. GSE41258 **b.** GSE28814 **c.** TCGA

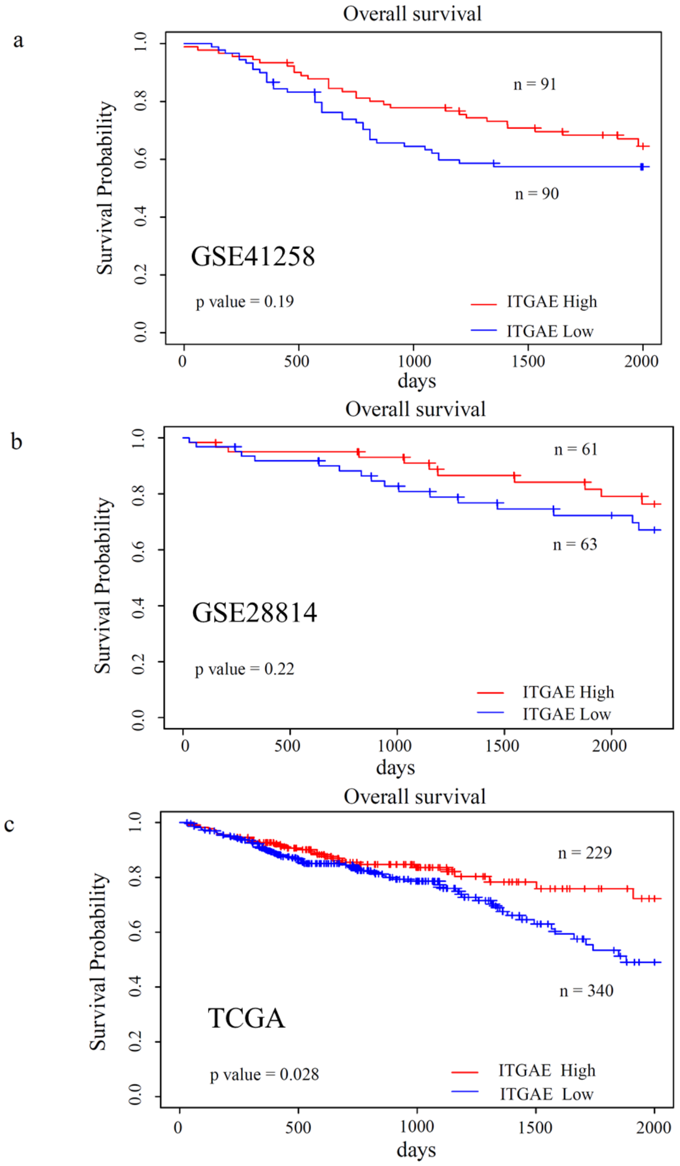

Supplementary Fig. 3. Immunohistochemical staining of infiltration CD103$^+$/CD8$^+$ lymphocytes with CD103 and CD8 antibodies in colorectal cancer

a. CD103$^+$ and CD8$^+$ cell counts were evaluated in serial sections. Representative images of highly infiltrated cases are shown on the left and lowly infiltrated cases are shown on the right. **b.** There was a significant correlation between CD103$^+$ and CD8$^+$ cell counts.

a

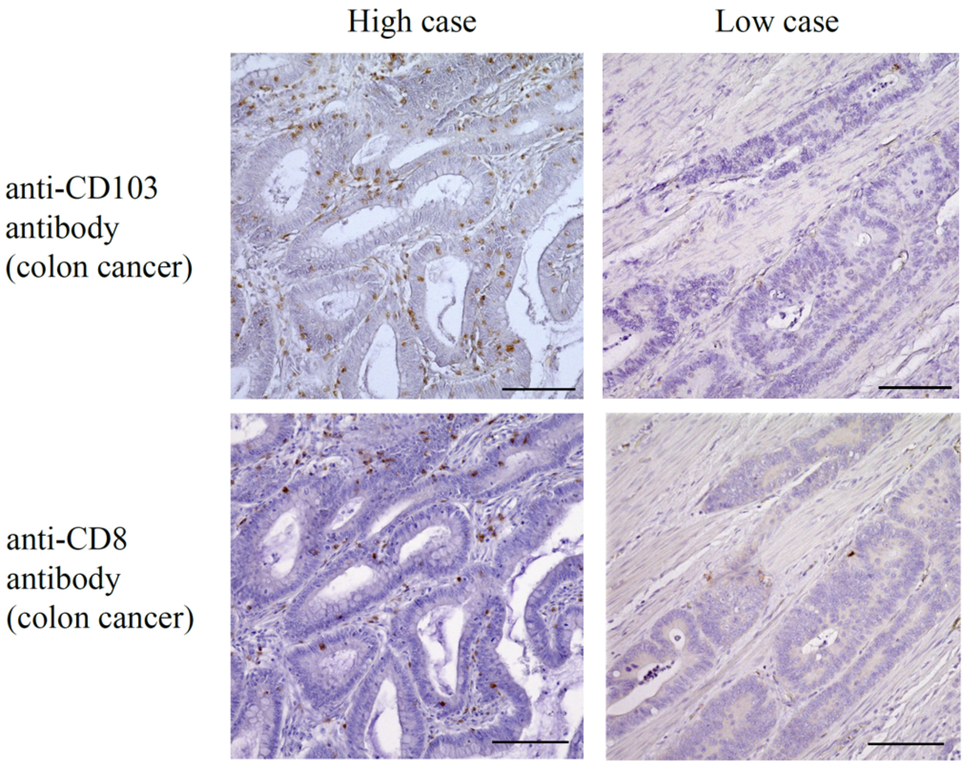

b

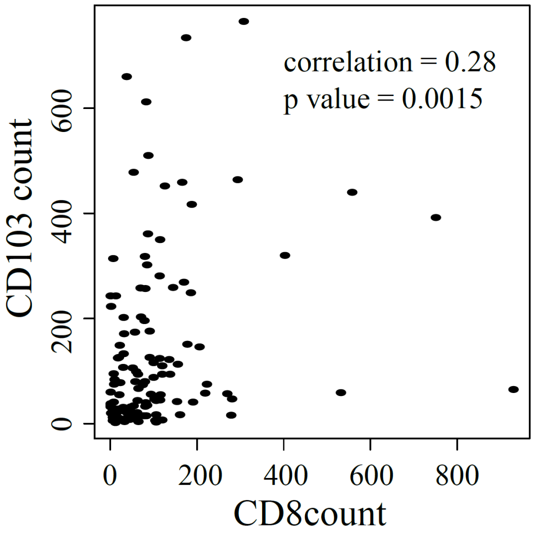

Supplementary Fig. 4. Number of CD103^{+} tumor-infiltrating lymphocytes (TILs), overall survival (OS) and recurrence-free survival (RFS)

a. The number of CD103^{+} cells and OS. **b.** The number of CD103^{+} cells and RFS. In the group with higher CD103^{+} cell count, both overall survival and relapse-free survival were significantly prolonged.

a

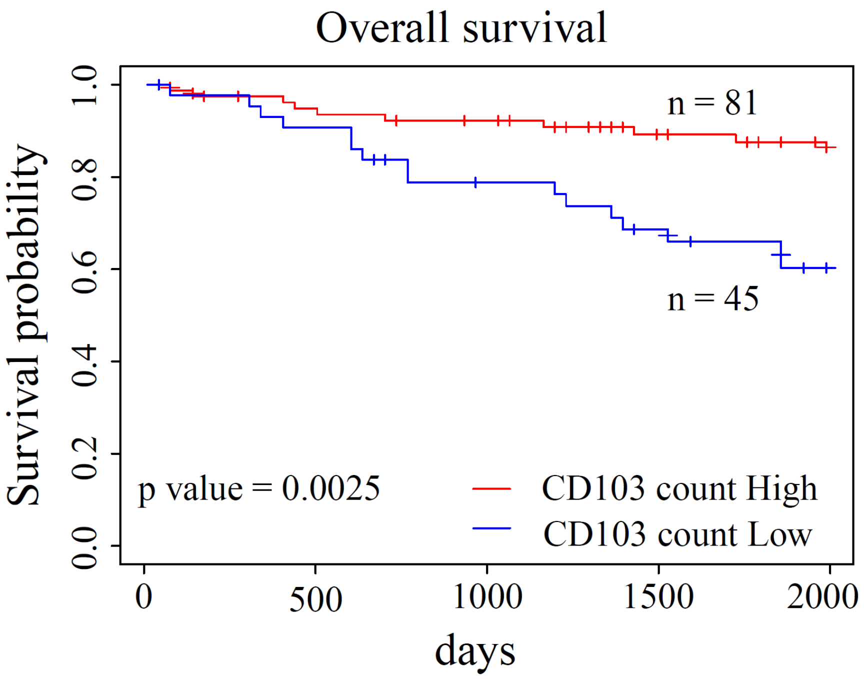

b

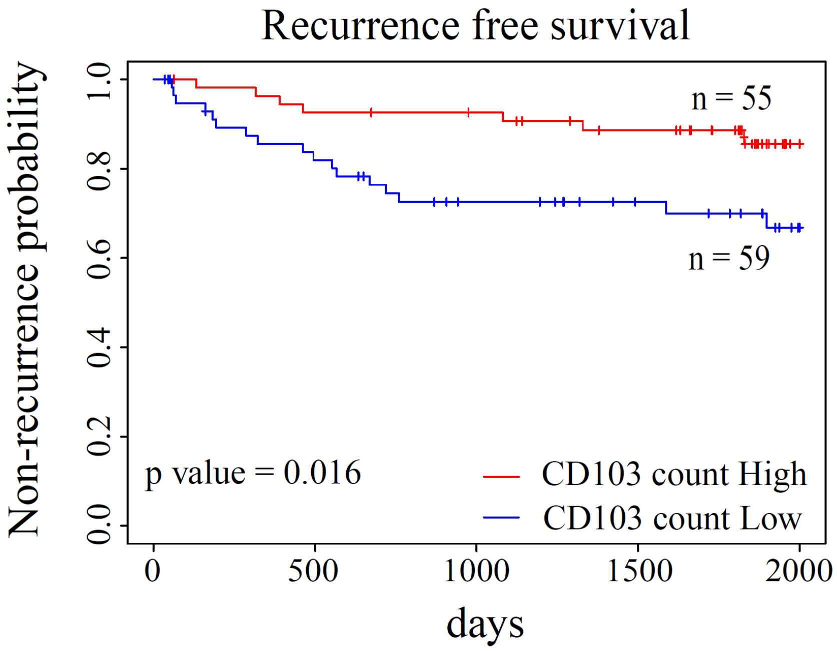

Supplementary Fig. 5. Comparison of CD8$^+$ and CD103$^+$ cell counts with pathological factors

a, c. The number of CD103-positive cells showed a significant relationship with lymphatic invasion and lymph node metastasis. **d.** In addition, both the CD8 and CD103 positive cell counts showed a significant relationship with distant metastasis.

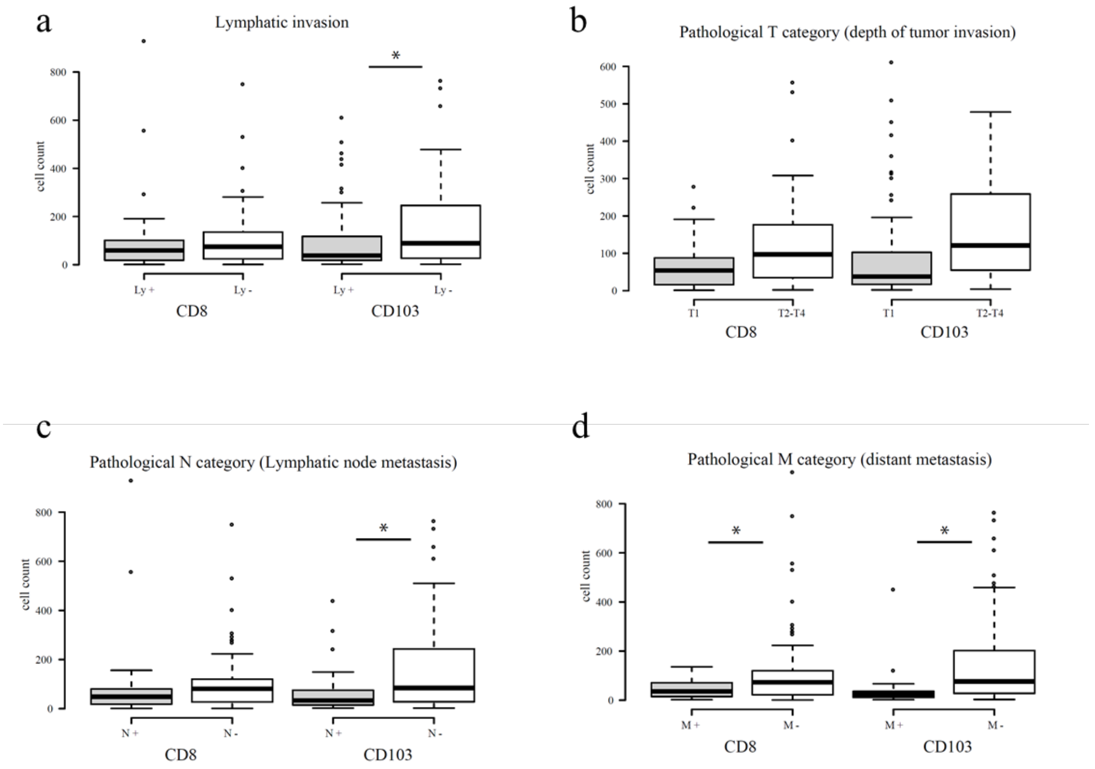

* $p < 0.05$

Supplementary Fig. 6. The TCR and IFNG signals and expression of ZNF683 in Trm in the GSE108989, GSE146771, and GSE164522 databases.

a. Gene expression distribution of Trm-related genes and cytotoxicity and exhaustion markers on TILs in colorectal cancer. **b.** Comparison of TBX21 and IFNG expression with that of ZNF683 in Trm. **c.** GSEA showed that compared with ZNF683+ Trm cells, ZNF683- Trm cells showed significantly higher TCR signaling and IFN-γ signaling. d. Schematic view of Trm cells in cancer tissue. TCR signaling induces cancer-specific Trm cells to express ZNF683, which in turn expresses IFNG. IFNG signaling induces TBX21 expression and positive feedback to ZNF683, which further enhances cytotoxicity.

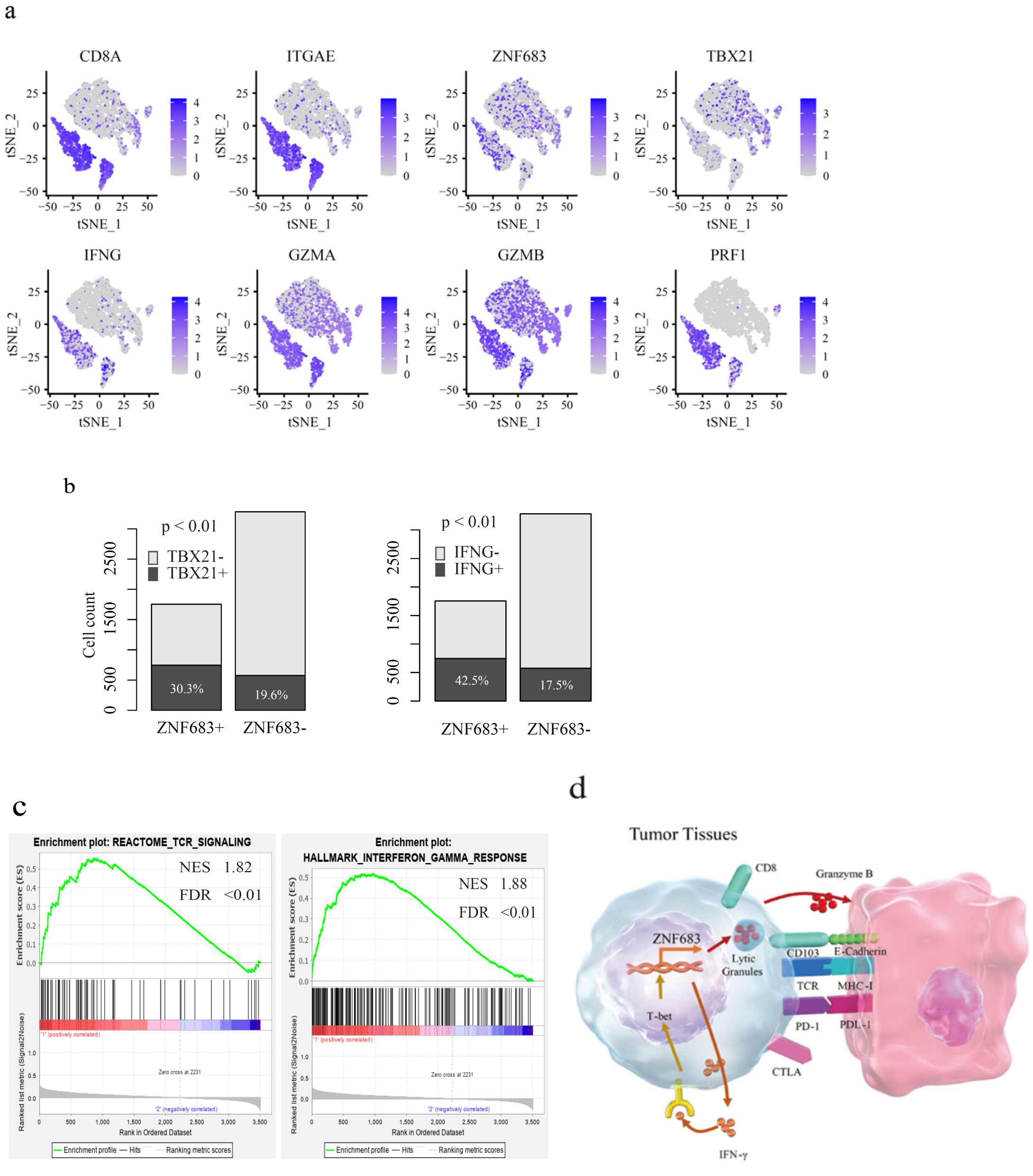

Supplementary Table 1. Baseline demographic and clinical characteristics of 126 patients with colorectal cancer.

	N=126
Age, median [year-old]	65（26- 87）
Sex [Male / Female]	77 / 49
BMI, median [kg/m^2]	22.3（16.4 – 34.2 ）
Location [colon / rectum]	70 / 56
Histological type [tub1 / tub2 / por, muc / NA]	61 / 51 / 11 / 3
Greatest diameter, median [mm]	35（3 – 135）
Preoperative CEA, median (ng/ml)	3（0.1-386）
Preoperative CA19-9, median (ng/ml)	15（1-15400)
Depth of invasion [T1 / T2 / T3 / T4 / NA]	47 / 19 / 49 / 9
Lymphatic invasion [+ / -]	63 / 63
Vascular invasion [+ / -]	28 / 98
Lymph node metastasis [+ / -]	38 / 88
Distant metastasis [+ / -]	16 / 110
Pathological Stage [0 / Ⅰ / Ⅱ / Ⅲ/ Ⅳ]	8 / 50 / 23 / 29 / 16
Number of CD103$^+$, median [cells/ 10HPF]	60（2-765）
Number of CD8$^+$, median [cells/10HPF]	62.5（1-930）

HPF, high power field

Supplementary Table 2. Comparison of patients' backgrounds between the high and low $CD8^+$/$CD103^+$ TILs in colorectal cancer

	HH $CD103^+$ (≧60/10HPF) and $CD8^+$ (≧ 62.5/10HPF)	LL $CD103^+$ (＜60/10HPF) and $CD8^+$ (＜ 62.5/10HPF)	P -value
Age [≧65/＜65]	25（61.0%）/16（39.0%）	19（46.3%）/22（53.7%）	0.268
Sex [male / female]	27（65.9%）/14（34.1%）	24（58.5%）/17（41.5%）	0.649
BMI [≦25kg/m^2/＞25kg/m^2]	36（87.8%）/5（12.2%）	31（81.6%）/7（18.4%）	0.537
Location [colon / rectum]	19（46.3%）/22（53.7%）	23（56.1%）/18（43.9%）	0.508
Histological type [tub1-2 / por, muc]	37（92.5%）/3（7.5%）	34（87.2%）/5（12.8%）	0.481
Greatest diameter [≧35 / ＜35] (mm)	20（48.8%）/21（51.2%）	28（73.7%）/10（26.3%）	0.037
Pathological T stage [T1 / T2-4]	22（53.7%）/19（46.3%）	8（20.0%）/32（80.0%）	0.002
Lymphatic invasion [+ / -]	19（46.3%）/22（53.7%）	26（66.7%）/13（33.3%）	0.076
Vascular invasion [+ / -]	8（19.5%）/33（80.5%）	12（30.8%）/27（69.2%）	0.306
Lymphatic node metastasis [+ / -]	9（22.0%）/32（88.0%）	19（47.5%）/21（52.5%）	0.020
Distant metastasis [+ / -]	3（7.3%）/38（92.7%）	12（29.3%）/29（70.7%）	0.020
Pathological Stage [Ⅰ / Ⅱ, Ⅲ, Ⅳ]	24（58.5%）/17（41.5%）	9（22.0%）/32（78.0%）	0.001

HPF, high power field

Supplementary Table 3. Factors evaluated for RFS (univariate and multivariate analyses).

**RFS**	**Univariate analysis**			**Multivariate analysis**		
**Variables**	**HR**	**95% CI**	***P*-value**	**HR**	**95% CI**	***P*-value**
Age (≧65 years)	1.25	0.60–2.63	0.551			
Sex (male)	1.27	0.55–2.74	0.546			
BMI (>25 kg/m^2)	1.39	0.48–4.00	0.546			
Tumor location (rectum)	1.40	0.67–2.95	0.372			
Histological type (Undifferentiated)	1.70	0.50–4.40	0.359			
Greatest tumor diameter (≧ 35 mm)	2.63	1.16–5.96	0.020	1.70	0.49–5.89	0.397
Pathological T category (T2–T4)	3.38	1.01–11.3	0.049	5.13	0.92–28.6	0.062
Lymphatic invasion (+)	2.47	1.12–5.45	0.025	1.90	0.53–6.90	0.327
Vascular invasion (+)	2.81	1.30–6.08	0.009	2.39	0.85–6.73	0.098
Lymph node metastasis (+)	1.68	0.79–3.56	0.180			
LL (CD103$^+$ <60 / 10HPF and CD8$^+$ <62 / 10HPF)	3.42	1.21–9.62	0.020	2.94	1.01–8.51	0.047

RFS, recurrence-free survival; HR, hazard ratio; CI, confidence interval; BMI, body mass index; LL, the numbers of both CD103$^+$ and CD8$^+$ cells were low; HPF, high power field

Supplementary Table 4. Clinical characteristics of 2 patients with colorectal cancer for single-call RNA-seq analysis.

	Case 1	Case2
Age [year-old]	80	54
Sex	Female	Male
Location	Colon	Rectum
Histological type	tub1	tub1>tub2
Greatest diameter, median [mm]	30	50
Depth of invasion	MP (T2)	SS (T3)
Lymph node metastasis	None	None
Distant metastasis	None	Liver metastasis
Pathological Stage	Stage I	Stage IV
Number of $CD103^{+}$, median [cells/ 10HPF]	350 (High)	31 (Low)
Number of $CD8^{+}$, median [cells/10HPF]	283 (High)	34 (Low)

HPF, high power field
