# Supplementary material for: Cancer-specific tissue-resident memory T-cells express ZNF683 in colorectal cancer
Source: Br J Cancer. 2023 Mar 3;128(10):1828–37. doi: 10.1038/s41416-023-02202-4 (PMC10147592; doi:10.1038/s41416-023-02202-4)
Supplement: Supplementary file 2 — authorship [file 41416_2023_2202_MOESM2_ESM.pdf]

<u>**Masatoshi Kitakaze**</u>

Dear Masatoshi Kitakaze:

We are very pleased to be informed that our manuscript BJC-A3339826R2 entitled "Cancer-specific tissue-resident memory T-cells express ZNF683 in colorectal cancer" has been **provisionally accepted** for publication in *British Journal of Cancer*.

Manuscript Number: BJC-A3339826R2
Title: Cancer-specific tissue-resident memory T-cells express ZNF683 in colorectal cancer
Authors: Masatoshi Kitakaze, Mamoru Uemura, Tomoaki Hara, Ryota Chijimatsu, Daisuke Motooka, Toshiro Hirai, Masamitsu Konno, Daisuke Okuzaki, Yuki Sekido, Tsuyoshi Hata, Takayuki Ogino, Hidekazu Takahashi, Norikatsu Miyoshi, Ken Ofusa, Tsunekazu Mizushima, Hidetoshi Eguchi, Yuichiro Doki, and Ishii Hideshi

To finalize the publication process, please check the final version of the manuscript and confirm that you agree with every change, such as title, authorship, text, tables, figures, and supplementary information.

We need your response as following:
Please put YES in the parenthesis.

Name: [Masatoshi Kitakaze], Date January 31, 2023

( ) I am agree with that final version of our manuscript BJC-A3339826R2 entitled "Cancer-specific tissue-resident memory T-cells express ZNF683 in colorectal cancer"

( ) I am disagree with that final version of our manuscript BJC-A3339826R2 entitled "Cancer-specific tissue-resident memory T-cells express ZNF683 in colorectal cancer”
Please indicate the reason why: (   )

Thank you very much in advance.

Sincerely,

Hideshi Ishii

＝＝＝＞REPLY

Name: [Masatoshi Kitakaze], Date January 31, 2023

(yes) I am agree with that final version of our manuscript BJC-A3339826R2 entitled "Cancer-specific tissue-resident memory T-cells express ZNF683 in colorectal cancer"

( ) I am disagree with that final version of our manuscript BJC-A3339826R2 entitled "Cancer-specific tissue-resident memory T-cells express ZNF683 in colorectal cancer”

Please indicate the reason why: (   )

Mamoru Uemura

Dear Mamoru Uemura:

We are very pleased to be informed that our manuscript BJC-A3339826R2 entitled "Cancer-specific tissue-resident memory T-cells express ZNF683 in colorectal cancer" has been **provisionally accepted** for publication in *British Journal of Cancer*.

Manuscript Number: BJC-A3339826R2
Title: Cancer-specific tissue-resident memory T-cells express ZNF683 in colorectal cancer
Authors: Masatoshi Kitakaze, Mamoru Uemura, Tomoaki Hara, Ryota Chijimatsu, Daisuke Motooka, Toshiro Hirai, Masamitsu Konno, Daisuke Okuzaki, Yuki Sekido, Tsuyoshi Hata, Takayuki Ogino, Hidekazu Takahashi, Norikatsu Miyoshi, Ken Ofusa, Tsunekazu Mizushima, Hidetoshi Eguchi, Yuichiro Doki, and Ishii Hideshi

To finalize the publication process, please check the final version of the manuscript and confirm that you agree with every change, such as title, authorship, text, tables, figures, and supplementary information.

We need your response as following:
Please put YES in the parenthesis.

Name: [Mamoru Uemura], Date January 31, 2023

( ) I am agree with that final version of our manuscript BJC-A3339826R2 entitled "Cancer-specific tissue-resident memory T-cells express ZNF683 in colorectal cancer"

( ) I am disagree with that final version of our manuscript BJC-A3339826R2 entitled "Cancer-specific tissue-resident memory T-cells express ZNF683 in colorectal cancer”
Please indicate the reason why: ( )

Thank you very much in advance.

Sincerely,

Hideshi Ishii

＝＝＝＞REPLY

Dear Prof. Ishii,

I am sending my response.

Name: [Mamoru Uemura], Date January 31, 2023

(**YES**) I am agree with that final version of our manuscript BJC-A3339826R2 entitled "Cancer-specific tissue-resident memory T-cells express ZNF683 in colorectal cancer"

( ) I am disagree with that final version of our manuscript BJC-A3339826R2 entitled "Cancer-specific tissue-resident memory T-cells express ZNF683 in colorectal cancer”
Please indicate the reason why: (   )

Best,

Mamoru Uemura

Tomoaki Hara

Dear Tomoaki Hara:

We are very pleased to be informed that our manuscript BJC-A3339826R2 entitled "Cancer-specific tissue-resident memory T-cells express ZNF683 in colorectal cancer" has been **provisionally accepted** for publication in *British Journal of Cancer*.

Manuscript Number: BJC-A3339826R2
Title: Cancer-specific tissue-resident memory T-cells express ZNF683 in colorectal cancer
Authors: Masatoshi Kitakaze, Mamoru Uemura, Tomoaki Hara, Ryota Chijimatsu, Daisuke Motooka, Toshiro Hirai, Masamitsu Konno, Daisuke Okuzaki, Yuki Sekido, Tsuyoshi Hata, Takayuki Ogino, Hidekazu Takahashi, Norikatsu Miyoshi, Ken Ofusa, Tsunekazu Mizushima, Hidetoshi Eguchi, Yuichiro Doki, and Ishii Hideshi

To finalize the publication process, please check the final version of the manuscript and confirm that you agree with every change, such as title, authorship, text, tables, figures, and supplementary information.

We need your response as following:
Please put YES in the parenthesis.

Name: [Tomoaki Hara], Date January 31, 2023

( ) I am agree with that final version of our manuscript BJC-A3339826R2 entitled "Cancer-specific tissue-resident memory T-cells express ZNF683 in colorectal cancer"

( ) I am disagree with that final version of our manuscript BJC-A3339826R2 entitled "Cancer-specific tissue-resident memory T-cells express ZNF683 in colorectal cancer”
Please indicate the reason why: ( )

Thank you very much in advance.

Sincerely,

Hideshi Ishii

＝＝＝>REPLY

Dear Professor Hideshi Ishii

We are very pleased to be informed that our manuscript BJC-A3339826R2 entitled "Cancer-specific tissue-resident memory T-cells express ZNF683 in colorectal cancer" has been **provisionally accepted** for publication in *British Journal of Cancer*.

Manuscript Number: BJC-A3339826R2
Title: Cancer-specific tissue-resident memory T-cells express ZNF683 in colorectal cancer
Authors: Masatoshi Kitakaze, Mamoru Uemura, Tomoaki Hara, Ryota Chijimatsu, Daisuke Motooka, Toshiro Hirai, Masamitsu Konno, Daisuke Okuzaki, Yuki Sekido, Tsuyoshi Hata, Takayuki Ogino, Hidekazu Takahashi, Norikatsu Miyoshi, Ken Ofusa, Tsunekazu Mizushima, Hidetoshi Eguchi, Yuichiro Doki, and Ishii Hideshi

To finalize the publication process, please check the final version of the manuscript and confirm that you agree with every change, such as title, authorship, text, tables, figures, and supplementary information.

We need your response as following:
Please put YES in the parenthesis.

Name: [Tomoaki Hara], Date January 31, 2023

(YES) I am agree with that final version of our manuscript BJC-A3339826R2 entitled ″Cancer-specific tissue-resident memory T-cells express ZNF683 in colorectal cancer″

( ) I am disagree with that final version of our manuscript BJC-A3339826R2 entitled ″Cancer-specific tissue-resident memory T-cells express ZNF683 in colorectal cancer”
Please indicate the reason why: ( )

Thank you very much in advance.

Sincerely,

Tomoaki Hara

Ryota Chijimatsu

Dear Ryota Chijimatsu:

We are very pleased to be informed that our manuscript BJC-A3339826R2 entitled "Cancer-specific tissue-resident memory T-cells express ZNF683 in colorectal cancer" has been **provisionally accepted** for publication in *British Journal of Cancer*.

Manuscript Number: BJC-A3339826R2
Title: Cancer-specific tissue-resident memory T-cells express ZNF683 in colorectal cancer
Authors: Masatoshi Kitakaze, Mamoru Uemura, Tomoaki Hara, Ryota Chijimatsu, Daisuke Motooka, Toshiro Hirai, Masamitsu Konno, Daisuke Okuzaki, Yuki Sekido, Tsuyoshi Hata, Takayuki Ogino, Hidekazu Takahashi, Norikatsu Miyoshi, Ken Ofusa, Tsunekazu Mizushima, Hidetoshi Eguchi, Yuichiro Doki, and Ishii Hideshi

To finalize the publication process, please check the final version of the manuscript and confirm that you agree with every change, such as title, authorship, text, tables, figures, and supplementary information.

We need your response as following:
Please put YES in the parenthesis.

Name: [Ryota Chijimatsu], Date January 31, 2023

( ) I am agree with that final version of our manuscript BJC-A3339826R2 entitled "Cancer-specific tissue-resident memory T-cells express ZNF683 in colorectal cancer"

( ) I am disagree with that final version of our manuscript BJC-A3339826R2 entitled "Cancer-specific tissue-resident memory T-cells express ZNF683 in colorectal cancer"
Please indicate the reason why: ( )

Thank you very much in advance.

Sincerely,

Hideshi Ishii

＝＝＝＞REPLY

Name: [Ryota Chijimatsu], Date January 31, 2023

(YES) I am agree with that final version of our manuscript BJC-A3339826R2 entitled "Cancer-specific tissue-resident memory T-cells express ZNF683 in colorectal cancer"

<u>Daisuke Motooka</u>

Dear Daisuke Motooka:

We are very pleased to be informed that our manuscript BJC-A3339826R2 entitled "Cancer-specific tissue-resident memory T-cells express ZNF683 in colorectal cancer" has been **provisionally accepted** for publication in *British Journal of Cancer*.

Manuscript Number: BJC-A3339826R2
Title: Cancer-specific tissue-resident memory T-cells express ZNF683 in colorectal cancer
Authors: Masatoshi Kitakaze, Mamoru Uemura, Tomoaki Hara, Ryota Chijimatsu, Daisuke Motooka, Toshiro Hirai, Masamitsu Konno, Daisuke Okuzaki, Yuki Sekido, Tsuyoshi Hata, Takayuki Ogino, Hidekazu Takahashi, Norikatsu Miyoshi, Ken Ofusa, Tsunekazu Mizushima, Hidetoshi Eguchi, Yuichiro Doki, and Ishii Hideshi

To finalize the publication process, please check the final version of the manuscript and confirm that you agree with every change, such as title, authorship, text, tables, figures, and supplementary information.

We need your response as following:
Please put YES in the parenthesis.

Name: [Daisuke Motooka], Date January 31, 2023

( ) I am agree with that final version of our manuscript BJC-A3339826R2 entitled "Cancer-specific tissue-resident memory T-cells express ZNF683 in colorectal cancer"

( ) I am disagree with that final version of our manuscript BJC-A3339826R2 entitled "Cancer-specific tissue-resident memory T-cells express ZNF683 in colorectal cancer”
Please indicate the reason why: (   )

Thank you very much in advance.

Sincerely,

Hideshi Ishii

＝＝＝＞REPLY

Dear Prof. Ishii,

Congratulations on the provisional acceptance of our manuscript. I am very pleased with the news.

I have reviewed the final version of the manuscript, BJC-A3339826R2 entitled "Cancer-specific tissue-resident memory T-cells express ZNF683 in colorectal cancer"
and I agree with all the changes including the title, authorship, text, tables, figures, and supplementary information.

Name: Daisuke Motooka, Date January 31, 2023

(YES) I am agree with the final version of our manuscript BJC-A3339826R2 entitled "Cancer-specific tissue-resident memory T-cells express ZNF683 in colorectal cancer".

Thank you for including me as one of the authors of this manuscript.

Sincerely yours,

Daisuke Motooka

Toshiro Hirai

Dear Toshiro Hirai:

We are very pleased to be informed that our manuscript BJC-A3339826R2 entitled "Cancer-specific tissue-resident memory T-cells express ZNF683 in colorectal cancer" has been **provisionally accepted** for publication in *British Journal of Cancer*.

Manuscript Number: BJC-A3339826R2
Title: Cancer-specific tissue-resident memory T-cells express ZNF683 in colorectal cancer
Authors: Masatoshi Kitakaze, Mamoru Uemura, Tomoaki Hara, Ryota Chijimatsu, Daisuke Motooka, Toshiro Hirai, Masamitsu Konno, Daisuke Okuzaki, Yuki Sekido, Tsuyoshi Hata, Takayuki Ogino, Hidekazu Takahashi, Norikatsu Miyoshi, Ken Ofusa, Tsunekazu Mizushima, Hidetoshi Eguchi, Yuichiro Doki, and Ishii Hideshi

To finalize the publication process, please check the final version of the manuscript and confirm that you agree with every change, such as title, authorship, text, tables, figures, and supplementary information.

We need your response as following:
Please put YES in the parenthesis.

Name: [Toshiro Hirai], Date January 31, 2023

( ) I am agree with that final version of our manuscript BJC-A3339826R2 entitled "Cancer-specific tissue-resident memory T-cells express ZNF683 in colorectal cancer"

( ) I am disagree with that final version of our manuscript BJC-A3339826R2 entitled "Cancer-specific tissue-resident memory T-cells express ZNF683 in colorectal cancer”
Please indicate the reason why: (   )

Thank you very much in advance.

Sincerely,

Hideshi Ishii

＝＝＝＞REPLY

Name: [Toshiro Hirai], Date January 31, 2023

(YES) I am agree with that final version of our manuscript BJC-A3339826R2 entitled ″Cancer-specific tissue-resident memory T-cells express ZNF683 in colorectal cancer″

( ) I am disagree with that final version of our manuscript BJC-A3339826R2 entitled ″Cancer-specific tissue-resident memory T-cells express ZNF683 in colorectal cancer”
Please indicate the reason why: (   )

Thank you very much in advance.

Sincerely,

Hideshi Ishii

Toshiro Hirai, Ph.D.
Vaccine Creation Group, BIKEN Innovative Vaccine Research Alliance Laboratories, Institute for Open and Transdisciplinary Research Initiatives/Research Institute for Microbial Diseases,
Osaka University
3-1 Yamadaoka, Suita, Osaka 565-0871, Japan
Tel: +81-6-6877-4920
Email: t-hirai@biken.osaka-u.ac.jp

<u>Masamitsu Konno</u>

Dear Masamitsu Konno:

We are very pleased to be informed that our manuscript BJC-A3339826R2 entitled "Cancer-specific tissue-resident memory T-cells express ZNF683 in colorectal cancer" has been **provisionally accepted** for publication in *British Journal of Cancer*.

Manuscript Number: BJC-A3339826R2
Title: Cancer-specific tissue-resident memory T-cells express ZNF683 in colorectal cancer
Authors: Masatoshi Kitakaze, Mamoru Uemura, Tomoaki Hara, Ryota Chijimatsu, Daisuke Motooka, Toshiro Hirai, Masamitsu Konno, Daisuke Okuzaki, Yuki Sekido, Tsuyoshi Hata, Takayuki Ogino, Hidekazu Takahashi, Norikatsu Miyoshi, Ken Ofusa, Tsunekazu Mizushima, Hidetoshi Eguchi, Yuichiro Doki, and Ishii Hideshi

To finalize the publication process, please check the final version of the manuscript and confirm that you agree with every change, such as title, authorship, text, tables, figures, and supplementary information.

We need your response as following:
Please put YES in the parenthesis.

Name: [Masamitsu Konno], Date January 31, 2023

( ) I am agree with that final version of our manuscript BJC-A3339826R2 entitled "Cancer-specific tissue-resident memory T-cells express ZNF683 in colorectal cancer"

( ) I am disagree with that final version of our manuscript BJC-A3339826R2 entitled "Cancer-specific tissue-resident memory T-cells express ZNF683 in colorectal cancer”
Please indicate the reason why: (   )

Thank you very much in advance.

Sincerely,

Hideshi Ishii

＝＝＝＞REPLY

Dear prof. Hideshi Ishii

Yes I am agree with that final version of our manuscript BJC-A3339826R2 entitled "Cancer-specific tissue-resident memory T-cells express ZNF683 in colorectal cancer”

Masamitsu Konno, Date January 31, 2023

<u>Daisuke Okuzaki</u>

Dear Daisuke Okuzaki:

We are very pleased to be informed that our manuscript BJC-A3339826R2 entitled "Cancer-specific tissue-resident memory T-cells express ZNF683 in colorectal cancer" has been **provisionally accepted** for publication in *British Journal of Cancer*.

Manuscript Number: BJC-A3339826R2
Title: Cancer-specific tissue-resident memory T-cells express ZNF683 in colorectal cancer
Authors: Masatoshi Kitakaze, Mamoru Uemura, Tomoaki Hara, Ryota Chijimatsu, Daisuke Motooka, Toshiro Hirai, Masamitsu Konno, Daisuke Okuzaki, Yuki Sekido, Tsuyoshi Hata, Takayuki Ogino, Hidekazu Takahashi, Norikatsu Miyoshi, Ken Ofusa, Tsunekazu Mizushima, Hidetoshi Eguchi, Yuichiro Doki, and Ishii Hideshi

To finalize the publication process, please check the final version of the manuscript and confirm that you agree with every change, such as title, authorship, text, tables, figures, and supplementary information.

We need your response as following:
Please put YES in the parenthesis.

Name: [Daisuke Okuzaki], Date January 31, 2023

( ) I am agree with that final version of our manuscript BJC-A3339826R2 entitled "Cancer-specific tissue-resident memory T-cells express ZNF683 in colorectal cancer"

( ) I am disagree with that final version of our manuscript BJC-A3339826R2 entitled "Cancer-specific tissue-resident memory T-cells express ZNF683 in colorectal cancer"
Please indicate the reason why: ( )

Thank you very much in advance.

Sincerely,

Hideshi Ishii

＝＝＝＞REPLY

Dear prof. Hideshi Ishii,

Congratulations on the provisional acceptance of your paper. Please find my response below for your review.

(YES) I am agree with that final version of our manuscript BJC-A3339826R2 entitled "Cancer-specific tissue-resident memory T-cells express ZNF683 in colorectal cancer"

Best regards,

Daisuke Okuzaki

Yuki Sekido

Dear Yuki Sekido:

We are very pleased to be informed that our manuscript BJC-A3339826R2 entitled "Cancer-specific tissue-resident memory T-cells express ZNF683 in colorectal cancer" has been **provisionally accepted** for publication in *British Journal of Cancer*.

Manuscript Number: BJC-A3339826R2
Title: Cancer-specific tissue-resident memory T-cells express ZNF683 in colorectal cancer
Authors: Masatoshi Kitakaze, Mamoru Uemura, Tomoaki Hara, Ryota Chijimatsu, Daisuke Motooka, Toshiro Hirai, Masamitsu Konno, Daisuke Okuzaki, Yuki Sekido, Tsuyoshi Hata, Takayuki Ogino, Hidekazu Takahashi, Norikatsu Miyoshi, Ken Ofusa, Tsunekazu Mizushima, Hidetoshi Eguchi, Yuichiro Doki, and Ishii Hideshi

To finalize the publication process, please check the final version of the manuscript and confirm that you agree with every change, such as title, authorship, text, tables, figures, and supplementary information.

We need your response as following:
Please put YES in the parenthesis.

Name: [Yuki Sekido], Date January 31, 2023

( ) I am agree with that final version of our manuscript BJC-A3339826R2 entitled "Cancer-specific tissue-resident memory T-cells express ZNF683 in colorectal cancer"

( ) I am disagree with that final version of our manuscript BJC-A3339826R2 entitled "Cancer-specific tissue-resident memory T-cells express ZNF683 in colorectal cancer”
Please indicate the reason why: (   )

Thank you very much in advance.

Sincerely,

Hideshi Ishii

＝＝＝＞REPLY

Name: [Yuki Sekido], Date January 31, 2023

(YES) I am agree with that final version of our manuscript BJC-A3339826R2 entitled "Cancer-specific tissue-resident memory T-cells express ZNF683 in colorectal cancer"

<u>Tsuyoshi Hata</u>

Dear Tsuyoshi Hata:

We are very pleased to be informed that our manuscript BJC-A3339826R2 entitled "Cancer-specific tissue-resident memory T-cells express ZNF683 in colorectal cancer" has been **provisionally accepted** for publication in *British Journal of Cancer*.

Manuscript Number: BJC-A3339826R2
Title: Cancer-specific tissue-resident memory T-cells express ZNF683 in colorectal cancer
Authors: Masatoshi Kitakaze, Mamoru Uemura, Tomoaki Hara, Ryota Chijimatsu, Daisuke Motooka, Toshiro Hirai, Masamitsu Konno, Daisuke Okuzaki, Yuki Sekido, Tsuyoshi Hata, Takayuki Ogino, Hidekazu Takahashi, Norikatsu Miyoshi, Ken Ofusa, Tsunekazu Mizushima, Hidetoshi Eguchi, Yuichiro Doki, and Ishii Hideshi

To finalize the publication process, please check the final version of the manuscript and confirm that you agree with every change, such as title, authorship, text, tables, figures, and supplementary information.

We need your response as following:
Please put YES in the parenthesis.

Name: [Tsuyoshi Hata], Date January 31, 2023

( ) I am agree with that final version of our manuscript BJC-A3339826R2 entitled "Cancer-specific tissue-resident memory T-cells express ZNF683 in colorectal cancer"

( ) I am disagree with that final version of our manuscript BJC-A3339826R2 entitled "Cancer-specific tissue-resident memory T-cells express ZNF683 in colorectal cancer”
Please indicate the reason why: (   )

Thank you very much in advance.

Sincerely,

Hideshi Ishii

＝＝＝>REPLY

--

Name: [Tsuyoshi Hata], Date January 31, 2023

(YES) I am agree with that final version of our manuscript BJC-A3339826R2 entitled "Cancer-specific tissue-resident memory T-cells express ZNF683 in colorectal cancer"

( ) I am disagree with that final version of our manuscript BJC-A3339826R2 entitled "Cancer-specific tissue-resident memory T-cells express ZNF683 in colorectal cancer”

Please indicate the reason why: ( )

--

Takayuki Ogino

Dear Takayuki Ogino:

We are very pleased to be informed that our manuscript BJC-A3339826R2 entitled "Cancer-specific tissue-resident memory T-cells express ZNF683 in colorectal cancer" has been **provisionally accepted** for publication in *British Journal of Cancer*.

Manuscript Number: BJC-A3339826R2
Title: Cancer-specific tissue-resident memory T-cells express ZNF683 in colorectal cancer
Authors: Masatoshi Kitakaze, Mamoru Uemura, Tomoaki Hara, Ryota Chijimatsu, Daisuke Motooka, Toshiro Hirai, Masamitsu Konno, Daisuke Okuzaki, Yuki Sekido, Tsuyoshi Hata, Takayuki Ogino, Hidekazu Takahashi, Norikatsu Miyoshi, Ken Ofusa, Tsunekazu Mizushima, Hidetoshi Eguchi, Yuichiro Doki, and Ishii Hideshi

To finalize the publication process, please check the final version of the manuscript and confirm that you agree with every change, such as title, authorship, text, tables, figures, and supplementary information.

We need your response as following:
Please put YES in the parenthesis.

Name: [Takayuki Ogino], Date January 31, 2023

( ) I am agree with that final version of our manuscript BJC-A3339826R2 entitled "Cancer-specific tissue-resident memory T-cells express ZNF683 in colorectal cancer"

( ) I am disagree with that final version of our manuscript BJC-A3339826R2 entitled "Cancer-specific tissue-resident memory T-cells express ZNF683 in colorectal cancer"
Please indicate the reason why: (   )

Thank you very much in advance.

Sincerely,

Hideshi Ishii

＝＝＝＞REPLY

(YES) I am agree with that final version of our manuscript BJC-A3339826R2 entitled "Cancer-specific tissue-resident memory T-cells express ZNF683 in colorectal cancer"

Hidekazu Takahashi

Dear Hidekazu Takahashi:

We are very pleased to be informed that our manuscript BJC-A3339826R2 entitled "Cancer-specific tissue-resident memory T-cells express ZNF683 in colorectal cancer" has been **provisionally accepted** for publication in *British Journal of Cancer*.

Manuscript Number: BJC-A3339826R2
Title: Cancer-specific tissue-resident memory T-cells express ZNF683 in colorectal cancer
Authors: Masatoshi Kitakaze, Mamoru Uemura, Tomoaki Hara, Ryota Chijimatsu, Daisuke Motooka, Toshiro Hirai, Masamitsu Konno, Daisuke Okuzaki, Yuki Sekido, Tsuyoshi Hata, Takayuki Ogino, Hidekazu Takahashi, Norikatsu Miyoshi, Ken Ofusa, Tsunekazu Mizushima, Hidetoshi Eguchi, Yuichiro Doki, and Ishii Hideshi

To finalize the publication process, please check the final version of the manuscript and confirm that you agree with every change, such as title, authorship, text, tables, figures, and supplementary information.

We need your response as following:
Please put YES in the parenthesis.

Name: [Hidekazu Takahashi], Date January 31, 2023

( ) I am agree with that final version of our manuscript BJC-A3339826R2 entitled "Cancer-specific tissue-resident memory T-cells express ZNF683 in colorectal cancer"

( ) I am disagree with that final version of our manuscript BJC-A3339826R2 entitled "Cancer-specific tissue-resident memory T-cells express ZNF683 in colorectal cancer"
Please indicate the reason why: ( )

Thank you very much in advance.

Sincerely,

Hideshi Ishii

＝＝＝＞REPLY

Name: Hidekazu Takahashi, Date January 31, 2023

(YES) I am agree with that final version of our manuscript BJC-A3339826R2 entitled "Cancer-specific tissue-resident memory T-cells express ZNF683 in colorectal cancer"

( ) I am disagree with that final version of our manuscript BJC-A3339826R2 entitled "Cancer-specific tissue-resident memory T-cells express ZNF683 in colorectal cancer”
Please indicate the reason why: (   )

<u>**Norikatsu Miyoshi**</u>

Dear Norikatsu Miyoshi:

We are very pleased to be informed that our manuscript BJC-A3339826R2 entitled "Cancer-specific tissue-resident memory T-cells express ZNF683 in colorectal cancer" has been **provisionally accepted** for publication in *British Journal of Cancer*.

Manuscript Number: BJC-A3339826R2
Title: Cancer-specific tissue-resident memory T-cells express ZNF683 in colorectal cancer
Authors: Masatoshi Kitakaze, Mamoru Uemura, Tomoaki Hara, Ryota Chijimatsu, Daisuke Motooka, Toshiro Hirai, Masamitsu Konno, Daisuke Okuzaki, Yuki Sekido, Tsuyoshi Hata, Takayuki Ogino, Hidekazu Takahashi, Norikatsu Miyoshi, Ken Ofusa, Tsunekazu Mizushima, Hidetoshi Eguchi, Yuichiro Doki, and Ishii Hideshi

To finalize the publication process, please check the final version of the manuscript and confirm that you agree with every change, such as title, authorship, text, tables, figures, and supplementary information.

We need your response as following:
Please put YES in the parenthesis.

Name: [Norikatsu Miyoshi], Date January 31, 2023

( ) I am agree with that final version of our manuscript BJC-A3339826R2 entitled "Cancer-specific tissue-resident memory T-cells express ZNF683 in colorectal cancer"

( ) I am disagree with that final version of our manuscript BJC-A3339826R2 entitled "Cancer-specific tissue-resident memory T-cells express ZNF683 in colorectal cancer"
Please indicate the reason why: (   )

Thank you very much in advance.

Sincerely,

Hideshi Ishii

＝＝＝＞REPLY

We need your response as following:

Please put YES in the parenthesis.

Name: [Norikatsu Miyoshi], Date January 31, 2023

(Yes) I am agree with that final version of our manuscript BJC-A3339826R2 entitled "Cancer-specific tissue-resident memory T-cells express ZNF683 in colorectal cancer"

( ) I am disagree with that final version of our manuscript BJC-A3339826R2 entitled "Cancer-specific tissue-resident memory T-cells express ZNF683 in colorectal cancer”

Please indicate the reason why: ( )

Thank you very much in advance.

Ken Ofusa

Dear Ken Ofusa:

We are very pleased to be informed that our manuscript BJC-A3339826R2 entitled "Cancer-specific tissue-resident memory T-cells express ZNF683 in colorectal cancer" has been **provisionally accepted** for publication in *British Journal of Cancer*.

Manuscript Number: BJC-A3339826R2

Title: Cancer-specific tissue-resident memory T-cells express ZNF683 in colorectal cancer

Authors: Masatoshi Kitakaze, Mamoru Uemura, Tomoaki Hara, Ryota Chijimatsu, Daisuke Motooka, Toshiro Hirai, Masamitsu Konno, Daisuke Okuzaki, Yuki Sekido, Tsuyoshi Hata, Takayuki Ogino, Hidekazu Takahashi, Norikatsu Miyoshi, Ken Ofusa, Tsunekazu Mizushima, Hidetoshi Eguchi, Yuichiro Doki, and Ishii Hideshi

To finalize the publication process, please check the final version of the manuscript and confirm that you agree with every change, such as title, authorship, text, tables, figures, and supplementary information.

We need your response as following:

Please put YES in the parenthesis.

Name: [Ken Ofusa], Date January 31, 2023

( ) I am agree with that final version of our manuscript BJC-A3339826R2 entitled "Cancer-specific tissue-resident memory T-cells express ZNF683 in colorectal cancer"

( ) I am disagree with that final version of our manuscript BJC-A3339826R2 entitled "Cancer-specific tissue-resident memory T-cells express ZNF683 in colorectal cancer”

Please indicate the reason why: (   )

Thank you very much in advance.

Sincerely,

Hideshi Ishii

＝＝＝＞REPLY

Name: [Ken Ofusa], Date January 31, 2023

(YES) I am agree with that final version of our manuscript BJC-A3339826R2 entitled ″Cancer-specific tissue-resident memory T-cells express ZNF683 in colorectal cancer″

( ) I am disagree with that final version of our manuscript BJC-A3339826R2 entitled ″Cancer-specific tissue-resident memory T-cells express ZNF683 in colorectal cancer”
Please indicate the reason why: (   )

Tsunekazu Mizushima

Dear Tsunekazu Mizushima:

We are very pleased to be informed that our manuscript BJC-A3339826R2 entitled "Cancer-specific tissue-resident memory T-cells express ZNF683 in colorectal cancer" has been **provisionally accepted** for publication in *British Journal of Cancer*.

Manuscript Number: BJC-A3339826R2
Title: Cancer-specific tissue-resident memory T-cells express ZNF683 in colorectal cancer
Authors: Masatoshi Kitakaze, Mamoru Uemura, Tomoaki Hara, Ryota Chijimatsu, Daisuke Motooka, Toshiro Hirai, Masamitsu Konno, Daisuke Okuzaki, Yuki Sekido, Tsuyoshi Hata, Takayuki Ogino, Hidekazu Takahashi, Norikatsu Miyoshi, Ken Ofusa, Tsunekazu Mizushima, Hidetoshi Eguchi, Yuichiro Doki, and Ishii Hideshi

To finalize the publication process, please check the final version of the manuscript and confirm that you agree with every change, such as title, authorship, text, tables, figures, and supplementary information.

We need your response as following:
Please put YES in the parenthesis.

Name: [Tsunekazu Mizushima], Date January 31, 2023

( ) I am agree with that final version of our manuscript BJC-A3339826R2 entitled "Cancer-specific tissue-resident memory T-cells express ZNF683 in colorectal cancer"

( ) I am disagree with that final version of our manuscript BJC-A3339826R2 entitled "Cancer-specific tissue-resident memory T-cells express ZNF683 in colorectal cancer"
Please indicate the reason why: ( )

Thank you very much in advance.

Sincerely,

Hideshi Ishii

＝＝＝＞REPLY

Name: [Tsunekazu Mizushima], Date January 31, 2023

(Yes) I am agree with that final version of our manuscript BJC-A3339826R2 entitled "Cancer-specific tissue-resident memory T-cells express ZNF683 in colorectal cancer"

( ) I am disagree with that final version of our manuscript BJC-A3339826R2 entitled "Cancer-specific tissue-resident memory T-cells express ZNF683 in colorectal cancer”
Please indicate the reason why: ( )

Hidetoshi Eguchi

Dear Prof. Hidetoshi Eguchi:

We are very pleased to be informed that our manuscript BJC-A3339826R2 entitled "Cancer-specific tissue-resident memory T-cells express ZNF683 in colorectal cancer" has been **provisionally accepted** for publication in *British Journal of Cancer*.

Manuscript Number: BJC-A3339826R2
Title: Cancer-specific tissue-resident memory T-cells express ZNF683 in colorectal cancer
Authors: Masatoshi Kitakaze, Mamoru Uemura, Tomoaki Hara, Ryota Chijimatsu, Daisuke Motooka, Toshiro Hirai, Masamitsu Konno, Daisuke Okuzaki, Yuki Sekido, Tsuyoshi Hata, Takayuki Ogino, Hidekazu Takahashi, Norikatsu Miyoshi, Ken Ofusa, Tsunekazu Mizushima, Hidetoshi Eguchi, Yuichiro Doki, and Ishii Hideshi

To finalize the publication process, please check the final version of the manuscript and confirm that you agree with every change, such as title, authorship, text, tables, figures, and supplementary information.

We need your response as following:
Please put YES in the parenthesis.

Name: [Hidetoshi Eguchi], Date January 31, 2023

( ) I am agree with that final version of our manuscript BJC-A3339826R2 entitled "Cancer-specific tissue-resident memory T-cells express ZNF683 in colorectal cancer"

( ) I am disagree with that final version of our manuscript BJC-A3339826R2 entitled "Cancer-specific tissue-resident memory T-cells express ZNF683 in colorectal cancer”
Please indicate the reason why: ( )

Thank you very much in advance.

Sincerely,

Hideshi Ishii

＝＝＝＞REPLY

Name: [Hidetoshi Eguchi], Date January 31, 2023

(YES) I am agree with that final version of our manuscript BJC-A3339826R2 entitled ″Cancer-specific tissue-resident memory T-cells express ZNF683 in colorectal cancer″

( ) I am disagree with that final version of our manuscript BJC-A3339826R2 entitled ″Cancer-specific tissue-resident memory T-cells express ZNF683 in colorectal cancer”
Please indicate the reason why: (   )

<u>Yuichiro Doki</u>

Dear Prof. Yuichiro Doki:

We are very pleased to be informed that our manuscript BJC-A3339826R2 entitled "Cancer-specific tissue-resident memory T-cells express ZNF683 in colorectal cancer" has been **provisionally accepted** for publication in *British Journal of Cancer*.

Manuscript Number: BJC-A3339826R2
Title: Cancer-specific tissue-resident memory T-cells express ZNF683 in colorectal cancer
Authors: Masatoshi Kitakaze, Mamoru Uemura, Tomoaki Hara, Ryota Chijimatsu, Daisuke Motooka, Toshiro Hirai, Masamitsu Konno, Daisuke Okuzaki, Yuki Sekido, Tsuyoshi Hata, Takayuki Ogino, Hidekazu Takahashi, Norikatsu Miyoshi, Ken Ofusa, Tsunekazu Mizushima, Hidetoshi Eguchi, Yuichiro Doki, and Ishii Hideshi

To finalize the publication process, please check the final version of the manuscript and confirm that you agree with every change, such as title, authorship, text, tables, figures, and supplementary information.

We need your response as following:
Please put YES in the parenthesis.

Name: [Yuichiro Doki], Date January 31, 2023

( ) I am agree with that final version of our manuscript BJC-A3339826R2 entitled "Cancer-specific tissue-resident memory T-cells express ZNF683 in colorectal cancer"

( ) I am disagree with that final version of our manuscript BJC-A3339826R2 entitled "Cancer-specific tissue-resident memory T-cells express ZNF683 in colorectal cancer"
Please indicate the reason why: (   )

Thank you very much in advance.

Sincerely,

Hideshi Ishii

＝＝＝＞REPLY

Name: [Yuichiro Doki], Date January 31, 2023

(yes) I am agree with that final version of our manuscript BJC-A3339826R2 entitled ″Cancer-specific tissue-resident memory T-cells express ZNF683 in colorectal cancer″

( ) I am disagree with that final version of our manuscript BJC-A3339826R2 entitled ″Cancer-specific tissue-resident memory T-cells express ZNF683 in colorectal cancer”

Please indicate the reason why: ( )

Hideshi Ishii

Name: [HIDESHI ISHII], Date January 31, 2023

(yes) I am agree with that final version of our manuscript BJC-A3339826R2 entitled ″Cancer-specific tissue-resident memory T-cells express ZNF683 in colorectal cancer″

( ) I am disagree with that final version of our manuscript BJC-A3339826R2 entitled ″Cancer-specific tissue-resident memory T-cells express ZNF683 in colorectal cancer”
Please indicate the reason why: (   )
